# Applications of microscopy and small angle scattering techniques for the characterisation of supramolecular gels

**DOI:** 10.3762/bjoc.20.220

**Published:** 2024-10-16

**Authors:** Connor R M MacDonald, Emily R Draper

**Affiliations:** 1 School of Chemistry, University of Glasgow, Glasgow, Scotland, G12 8QQ, UK

**Keywords:** characterisation, electron microscopy, gelators, small angle scattering, supramolecular materials

## Abstract

When evaluating soft self-assembling materials for use in any application, the structural or morphological characterisation is highly important. We know that the hierarchal molecular self-assembly of these materials into larger structures directly influences behaviours such as performance and stability. It is therefore imperative that these materials are characterised effectively over multiple length scales. Two effective methods of achieving this are small angle scattering (SAS) and imaging. Scattering giving us indirect information about the systems, whereas imaging is often looking at the material directly. In this review, we discuss the benefits, caveats and power of using both these techniques separately and together for the characterisation of supramolecular gels.

## Introduction

**Figure 1:**
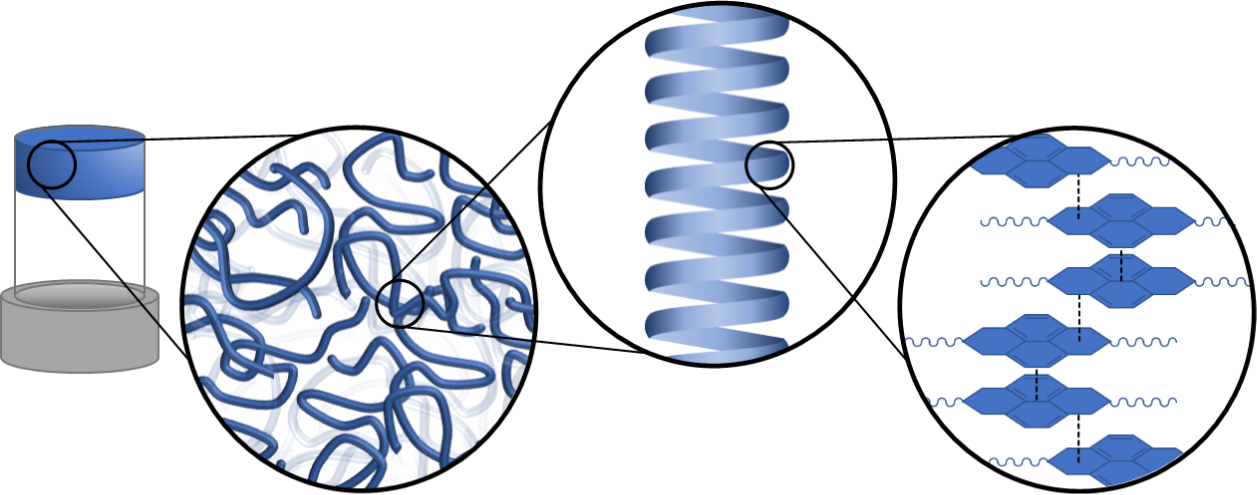
Hierarchical assembly occurring across length scales. Molecular interactions result in fibres which can form a network giving rise to the bulk gel.

Supramolecular gels are a versatile class of materials, and are of interest in many diverse applications from energy storage to cell culture [1–2]. The versatile nature of soft matter systems has been of interest to materials scientists for decades [3–5]. The large number of materials and processing techniques under development will allow these materials to be utilised in increasingly sophisticated applications such as drug delivery, environmental remediation, sensing, optoelectronics, photo-responsive actuators, and healable materials, and a range of biomedical applications among many others [6–9]. To better understand and guide the development of these chemical systems, robust characterisation techniques and protocols are required. These materials utilise the self-assembly of small molecules into a network of long anisotropic structures which can entangle or cross-link. This network can immobilise the solvent, resulting in a material with both solid-like and liquid-like characteristics. The versatility of these compounds arises from their self-assembly across many length scales (Figure 1), allowing for the control of properties through not only chemical modifications on the monomer, but also changes to the gelation process [2]. Whilst this tunability is advantageous in that it allows for a wide range of functionality and applications, this also gives rise to difficulties in understanding and predicting gel structure and properties [2]. In order to understand the supramolecular structure, a robust characterisation regime is required. A comprehensive work edited by Weiss and Térech discussed in detail the plethora of chemical systems, characterisation techniques, and applications of molecular gels; which still remains deeply insightful despite the rapid advancement of the field [10]. A more recent review by Yu et al. summarised a number of techniques used to characterise supramolecular materials [11]. Two of the most commonly used methods of characterisation of the larger structures present are microscopy and scattering techniques [12]. Across the literature, many investigations rely solely on one of these techniques alone to describe their system. However, both microscopy and scattering have limitations which may prevent a robust characterisation from being achieved. In this review, we aim to highlight and discuss a number of strengths and limitations provided by these techniques and will show how using these characterisation methods to complement each other can provide a much better understanding of self-assembling systems.

## Review

### Imaging techniques

Imaging is the most widely used and accessible technique for characterising these gel materials. Researchers are looking for the presence of fibrous structures, coils, entanglements, spherulites and other such features that can give them some insight into how the gel is formed in the network via the assembly of interlocking nanostructures and microstructures. Imaging is often inexpensive and accessible both in terms of access and interpretation of the data given. These images are attractive and give an instant visual summary of the network. Depending on the type of imaging used, multiple different length scales can be probed to identify how the individual fibres form and then entangle to form the gel network. In addition to showing the shape of the self-assembled structures, with the aid of software tools such as ImageJ [13], features such as the fibre width, fibre length, and pore sizes can be quantified [14]. However, measurements are limited to the snapshot captured by the image and require that the fibres are manually measured. This can be error-prone, inconsistent, and time consuming which can both require significant expertise to perform and limit the number of samples that can be feasibly analysed [15]. A recent review by Kubota et al. summarised both the spatiotemporal advantages and disadvantages, as well as the artefacts arising from sample preparation and imaging conditions in the microscopy of molecular self-assemblies [16]. When imaging supramolecular gels, atomic force microscopy (AFM), electron microscopy (EM) techniques including transmission electron microscopy (TEM), scanning electron microscopy (SEM), and scanning transmission electron microscopy (STEM), and confocal laser scanning microscopy (CLSM) are the most common techniques for imaging self-assembled structures. Some of the key features and capabilities of these techniques are summarised in Table 1.

**Table 1:** Comparison of imaging techniques for organic molecular assemblies.^a^

	CLSM	AFM/high-speed AFM	TEM/SEM/STEM	Cryo-TEM	Liquid cell EM

spatial resolution	200 nm	≤1–50 nm	1–10 nm	0.2–10 nm	≤3–30 nm
acquisition speed (frame^−1^)	1 s–1 min	100 ms–10 s			30 ms–1 s
sample environment and preparation	solution, fluorescence labelling	dried or solution, sample on substrate	dried, sample on grid, (stained with metal)	vitrified, sample on grid	solution, sample in liquid cell
artefacts	bleaching, laser toxicity, dye addition	tip, force, scanning	dehydration, beam damage	vitrification, ice crystal formation, beam damage	beam damage, diffusional constraint
advantage	in situ imaging, multicolour imaging, 3 D imaging by Z stack	in situ imaging, no staining, imaging with various parameters, high vertical resolution	high resolution	solves near-atomic structures (single particle analysis), snapshot	in situ imaging
limitation	low spatial resolution, Imaging restricted to fluorescence labels	restricted to surface	no in situ imaging	no in situ imaging, thin film needed	lower resolution than other EM techniques, thin liquid sample needed

^a^Table 1 was adapted from [16], R. Kubota et al., “Microscopic Imaging Techniques for Molecular Assemblies: Electron, Atomic Force, and Confocal Microscopies”, Chem. Rev., © 2021 American Chemical Society. This content is not subject to CC BY 4.0.

**Figure 2:**
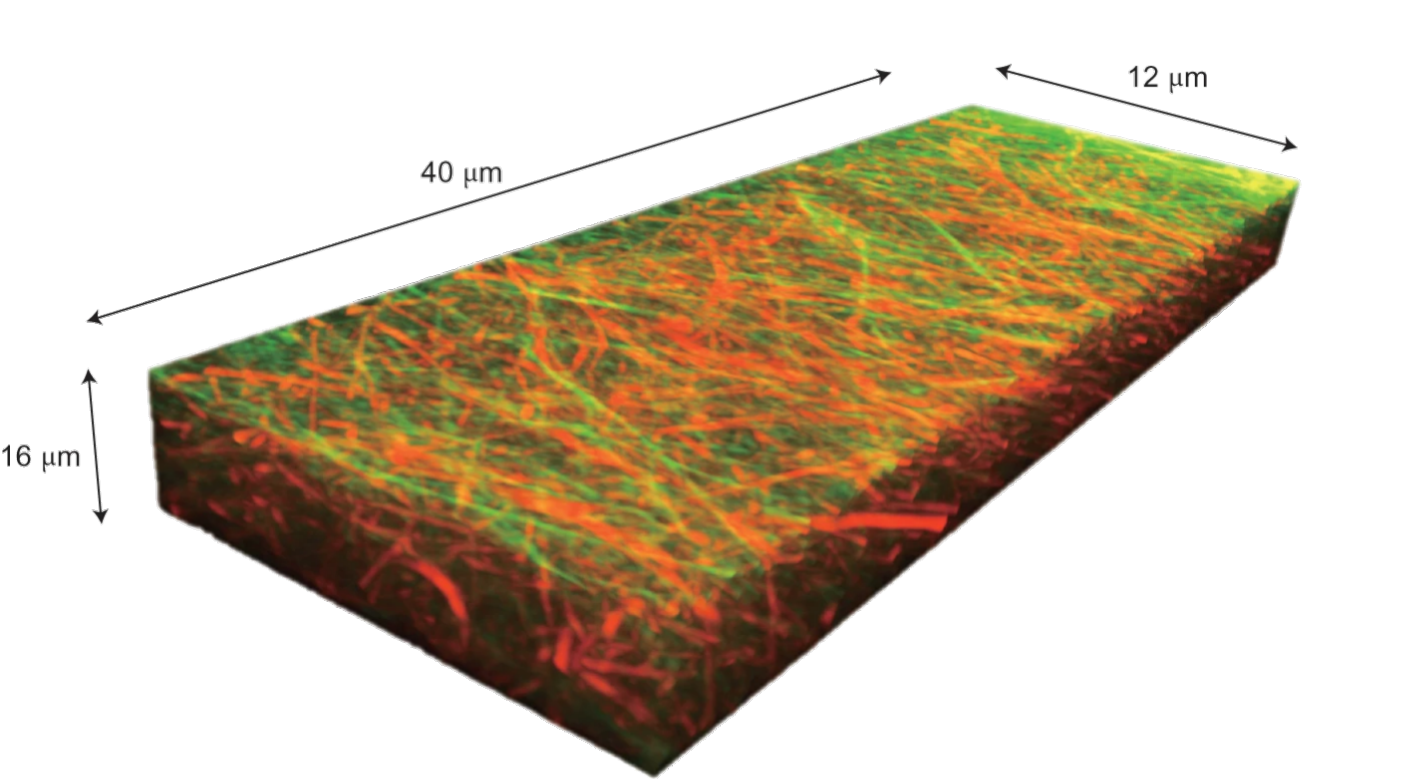
Three-dimensional CLSM image of a multicomponent supramolecular structure. The three-dimensional CLSM image is constructed from *z*-stacked *x*–*y* slice images. Figure 2 is from [17] and was adapted with permission from Springer Nature from the journal Nature Chemistry (“In situ real-time imaging of self-sorted supramolecular nanofibers” by S. Onogi; H. Shigemitsu; T. Yoshii; T. Tanida; M. Ikeda; R. Kubota; I. Hamachi), Copyright 2016, Springer Nature. This content is not subject to CC BY 4.0.

CLSM is a fluorescence microscopy technique capable of acquiring high-resolution optical images at selected depths of the sample. This is achieved by exciting compounds with laser light and, by using a pinhole to select focal planes, allows fluorescence at various depths to be focused and imaged. The resulting 2D focal plane images can be stacked to yield a 3D fluorescence image of high resolution, allowing complex morphologies to be visualised (Figure 2) [11].

**Figure 3:**
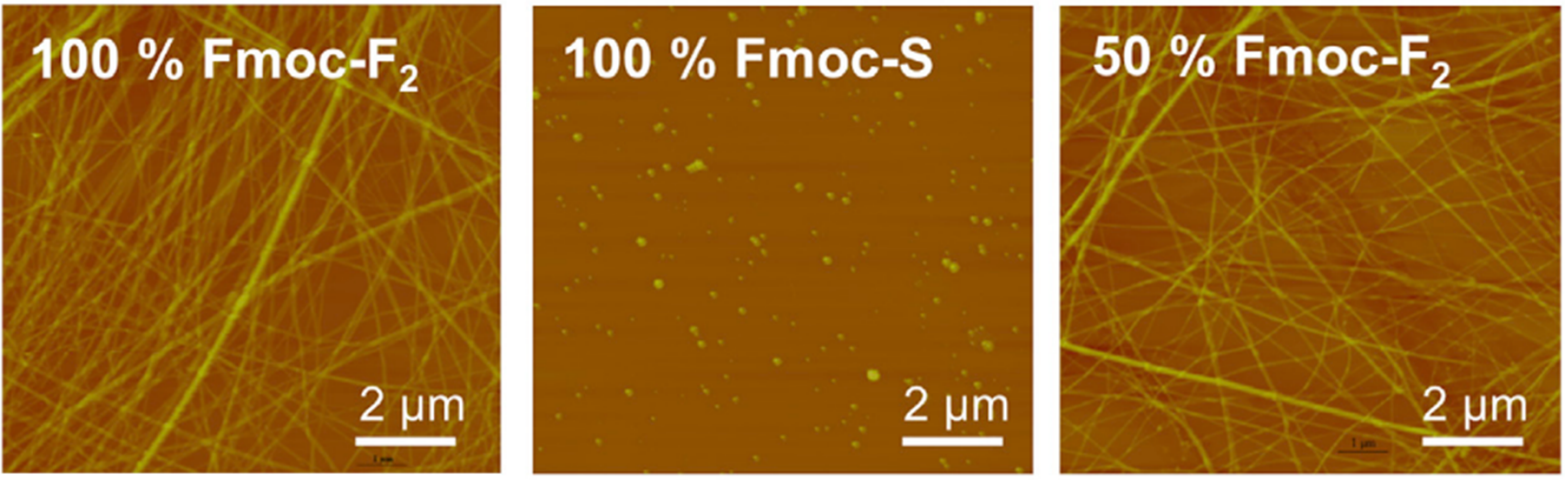
AFM images of air-dried aqueous Fmoc-FF, Fmoc-S, and 1:1 Fmoc-FF:Fmoc-S solutions. Figure 3 was reprinted from [20], Chem, vol. 1, by E. V. Alakpa; V. Jayawarna; A. Lampel; K. V. Burgess; C. C. West; S. C.J. Bakker; S. Roy; N. Javid; S. Fleming; D. A. Lamprou; J. Yang; A. Miller; A. J. Urquhart; P. W. J. M. Frederix; N. T. Hunt; B. Péault; R. V. Ulijn “Tunable Supramolecular Hydrogels for Selection of Lineage-Guiding Metabolites in Stem Cell Cultures”, pages 298–319, Copyright (2016), with permission from Elsevier. This content is not subject to CC BY 4.0.

While CLSM provides in situ 3D imaging, meaning no drying is required of the material, the material itself needs to be fluorescent (but not quenching upon assembly) or the addition of a dye is required. Such dyes need to be able to stick with the fibres, rather than sitting in the pores. Therefore, the fluorescent material is either chemically attached to the molecules themselves or is interacting with them. Introducing fluorescent dyes to self-assembling systems affects their chemical and mechanical stability as the hydrophobic features can destabilise the self-assembled network [18]. This reliance on fluorescence labels or dyes leading to a change in the self-assembly, meaning a true representation of the supramolecular material may not be investigated which is rarely confirmed when applied to the characterisation of such systems. To identify such changes in structure, other characterisation techniques that probe the structures without the use of fluorescence probes should be used in tandem with CLSM. For example, Raeburn et al. showed that the addition of molecular rotors and fluorescent dyes affects the bulk network of self-assembled materials using rheology. The co-packing was observed to alter rheological properties of the materials arising, for example, from differences in the rate of fibre growth and entanglements. The change in rheological properties when comparing the material and its co-assembly with the probe shows that addition of such probes may lead to measurements which do not truly represent the system of interest [19]. The work by Alakpa et al. highlights how important it is to consider how one assesses the impact of co-assembly on a molecular system [20]. AFM suggested that the addition of the surfactant-like Fmoc-serine (Fmoc-S) to the fibrous Fmoc-diphenylalanine (Fmoc-FF) did not impact the structure of the Fmoc-FF fibres (Figure 3). However, Fourier-transform infrared spectroscopy indicated that the Fmoc-S assembled on the surface of the Fmoc-FF fibres. The addition of carboxylate ions at the fibre surface allows cross-linking to occur, altering the bulk physical properties of the gel [20].

**Figure 4:**
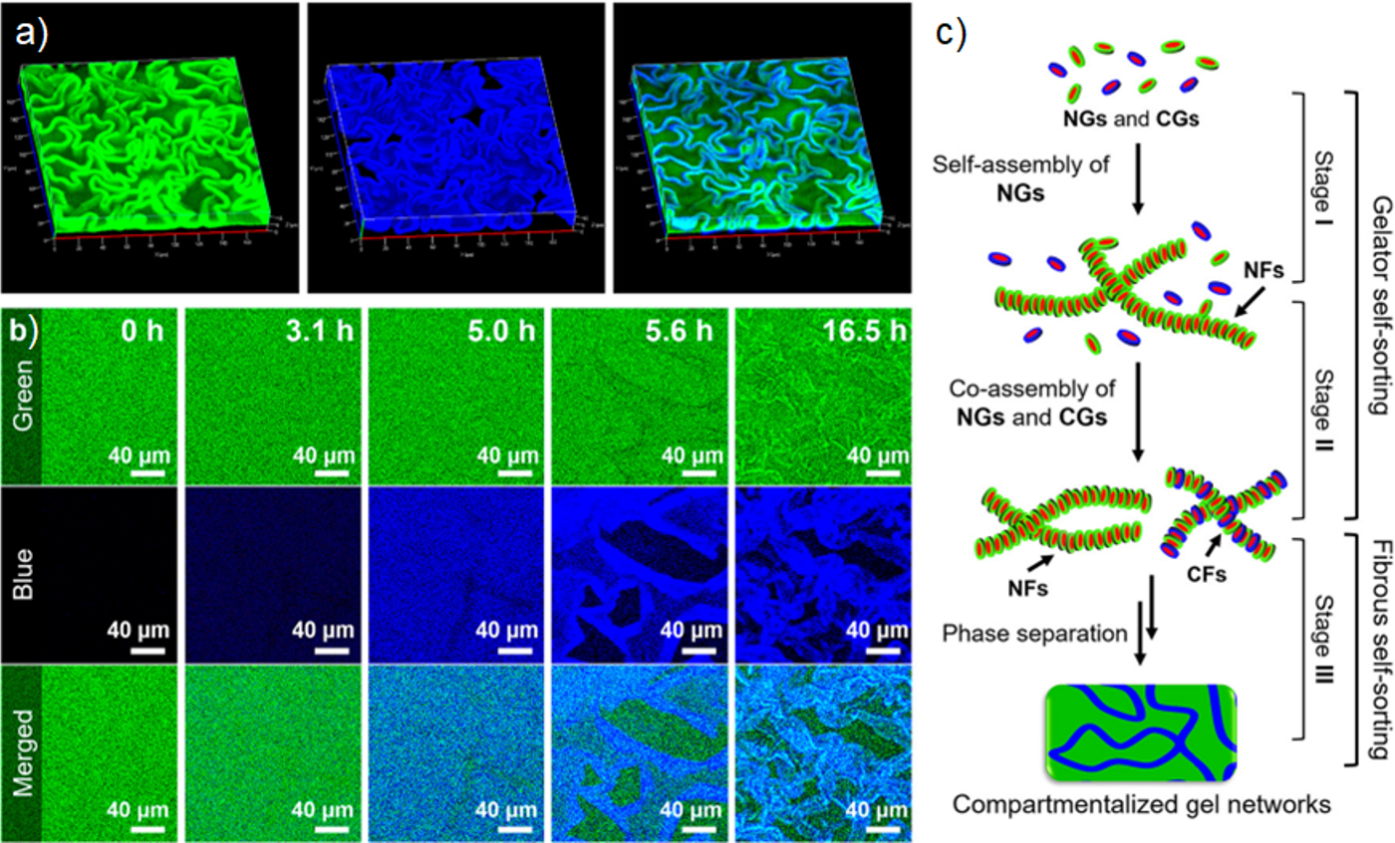
(a) 3D CLSM images of macroscopically a self-sorting gel network, where all fibres were stained green, but only CFs incorporated blue stain. (b) 2D CLSM images showing the formation and self-sorting of NFs and CFs over time. (c) Illustration of the multilevel self-sorting process. Figure 4 was adapted from [23], Y. Wang et al., “Hierarchically Compartmentalized Supramolecular Gels through Multilevel Self-Sorting”, J. Am. Chem. Soc., © 2018 American Chemical Society, distributed under the ACS AuthorChoice/Editors' Choice via CC-BY-NC-ND Usage Agreement, https://pubs.acs.org/page/policy/authorchoice_ccbyncnd_termsofuse.html. This content is not subject to CC BY 4.0.

While the proportions of fluorescent probes in the self-assembled material will be much smaller than the example discussed here, it is important to use appropriate methods to show that the addition of any co-assembling component does not impact the nanoscale structure and bulk network. To our knowledge there are very few examples in the literature that undertake analyses to show that the addition of a co-assembling probe does not disrupt the structure of the unmodified material. To adequately show that such adapted systems are relevant to the material of interest, future works should confirm that the modified systems are indeed representative. CLSM also often has poor resolution due to the use of light used for imaging. Other fluorescent imaging techniques such as super resolution fluorescence microscopy (for example, stimulated emission depletion (STED) microscopy or point accumulation for imaging in nanoscale topography (PAINT)) can give amazing details and resolution [17,21–22]. Techniques such as STED and PAINT require structures that are specifically designed and stable enough for the longer imaging times, and so they are often not suitable for many systems. Another advantage of these techniques is the possibility of using different probes in a multicomponent network. Onogi et al. reported one of the first examples of direct imaging, in situ, of self-sorted supramolecular nanofibres [17]. TEM was able to show that the mixture of gelators gave rise to nanofibres with a morphology similar to their single-component counterparts. The similarity between these systems made it difficult to assess whether the mixture of gelators resulted in self-sorted or co-assembled fibres using TEM alone. By developing distinct fluorescent probes which could selectively aggregate with, and subsequently stain the supramolecular fibres, CSLM was used to identify the mode of molecular assembly of each component. By comparing the CSLM images arising from the different fluorescent probes, there was shown to be a weak correlation between the resulting images, indicating that the fibres self-sort and separately entangle each other. By obtaining images in three dimensions, the orthogonal assembly could be directly visualised throughout the materials microstructure (Figure 2) [17]. This in situ method of CLSM also allows the kinetics of self-assembly to be elucidated, as demonstrated by Wang et al. [23]. By using fluorescent derivatives to visualise a multicomponent system consisting of neutral and charged gelators (NG and CG, respectively), the self-assembly of neutral and charged fibres (NF and CF, respectively) could be visualised over time. CSLM shows the self-assembly proceeds by the initial self-sorting of NGs to assemble NFs. The self-sorting of NFs decreases the concentration of NGs, increasing the relative concentration of CGs, allowing a higher critical assembly concentration of the CGs (arising from electrostatic repulsions between the CGs) to be reached, leading to the co-assembly of CFs. Not only could CLSM observe the initial kinetic self-sorting of gelators, but it also showed that the homogenous mixture of NFs and CFs can undergo a higher level of self-sorting, resulting in macroscopic phase separation (Figure 4).

**Figure 5:**
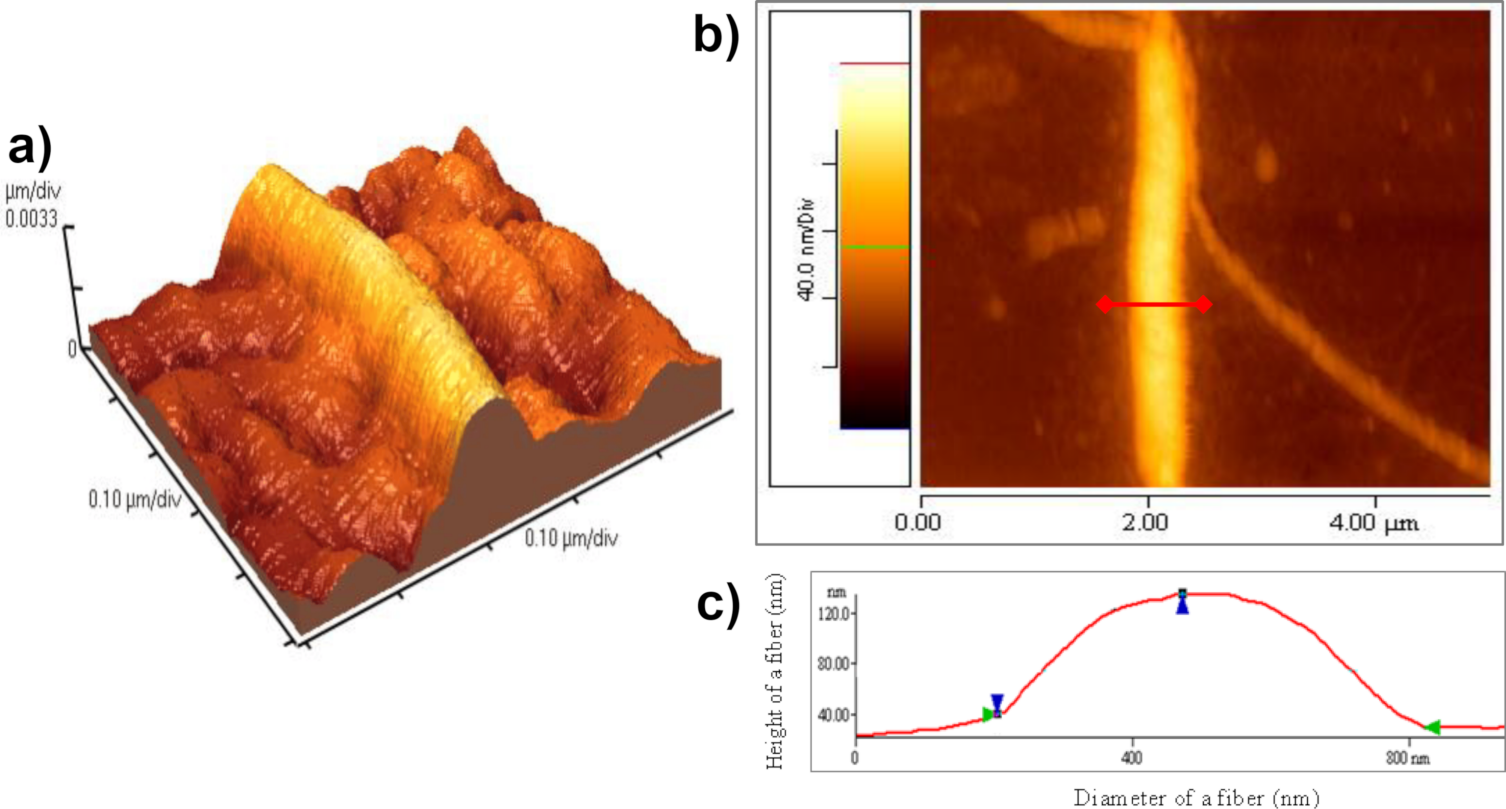
(a) 3D AFM topographic image of dried elastin fibre. (b) Indicative height and diameter profile plot of a fibre, with the red line representing the cross-section analysed in plot (c). Height and diameter of the fibre is calculated between the blue and green arrows, respectively. Figure 5 was adapted from [29] (© 2018 K. Sambani et al., published by MDPI, distributed under the terms of the Creative Commons Attribution 4.0 International License, https://creativecommons.org/licenses/by/4.0).

To gather a three-dimensional surface profile of a material, AFM utilises a highly sensitive cantilever to measure the nanometre to sub-millimetre surface morphology. Attractive or repulsive interactions between the surface and the cantilever induce a bending force in the cantilever providing a mechanical means of probing the surface nanostructure [11]. There are various modes of interaction available to AFM to probe the surface topology. The simplest is constant-height mode, wherein the sample is scanned with the cantilever at a constant height while measuring the deflection. Constant-height mode may result in large deflections and lead to sample damage by inducing lateral shear [24–25]. To minimise this, a constant-deflection mode can be employed by instead varying the cantilever height with a feedback loop to keep the deflection forces constant. The feedback output is used to generate a height image, however, often an imperfect feedback loop results in an error signal which generates a deflection image. The height image can be used to quantify the height and thickness of surface features, while the deflection image can probe fine surface details [24]. To effectively characterise soft materials, the minimisation of surface forces is essential. This fact made tapping-mode AFM, wherein the cantilever oscillated vertically to reduce the tip–sample contact, a revolutionary method for the characterisation of soft materials [25]. As well as probing the height of the surface, the phase-imaging afforded by the tapping-mode AFM can probe the viscoelastic properties and adhesion forces to qualitatively distinguish different materials in a sample [26–27]. By relying purely on the interactions between the cantilever and the material surface AFM can overcome the limitations of using fluorescent probes. However, by investigating only the surface of the materials, the bulk gel (which may not be the same as the surface) is inaccessible and cannot be characterised. AFM has the advantage of allowing for the measurement of hydrated samples, and therefore does not suffer from drying artefacts. Since hydrogels are mainly made of water and low concentration hydrogels can be very soft, AFM measurements face the problem of sample drift [28]. Avoiding drift artefacts is difficult so AFM is often performed in a dried state, but imaging of hydrogels in wet state has been achieved on thin films to minimise drift artefacts. Unlike EM techniques, AFM does not require treatments such as metal coating that can alter the structure of the material [11]. AFM was recently used by Sambani et al. to characterise the self-assembly of elastin nanofibres [29]. The high resolution imaging across length scales allows a range of sizes to be probed, identifying the fibres which arise from the formation of fibrils, which are themselves formed from nanofibrils (Figure 5). It is worth noting that difficulties arise in AFM with dealing with such differences in scale as an excessively large step height can result in artefacts that make it hard to detect smaller structures [29].

**Figure 6:**
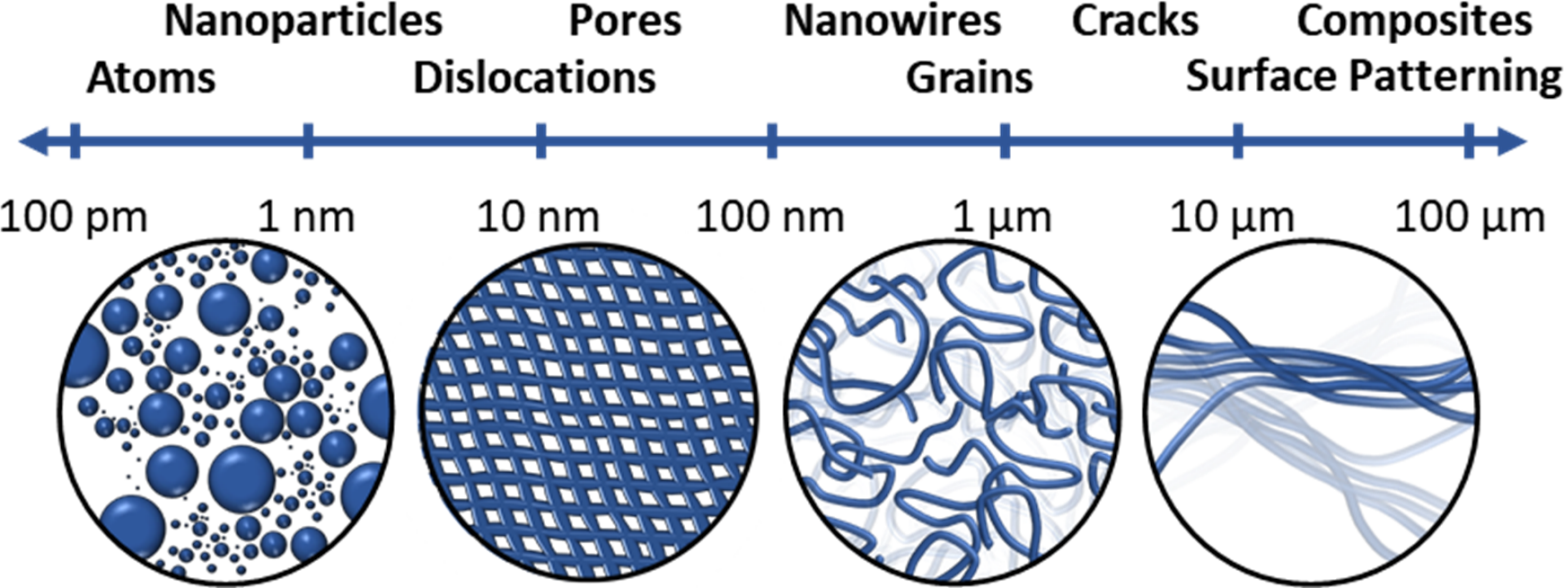
The nano-to-micro imaging range of SEM and TEM [30]. Cartoons represent the nanoparticles, pores, nanowires, and composites (from left to right, respectively) which can be probed by electron microscopy.

EM techniques, including scanning electron microscopy (SEM) and transmission electron microscopy (TEM), have proven to be some of the most robust and popular supramolecular characterisation techniques. SEM raster scans a fine beam of electrons over a sample surface. As electrons strike the surface, interactions between the beam and sample result in the emission of secondary scattered electrons, back-scattered electrons and X-rays which can be detected to produce an image. In contrast, TEM can measure the bright-field image, among other modes, by blocking the scattered electrons and detecting only the unscattered electrons. This results in a bright-field image where areas that are actively scattering have fewer electrons, resulting in a darker contrast [30]. Together SEM and TEM can enable multiscale quantification of nanostructural and microstructural features (Figure 6) [30].

The versatility of EM can reveal a number of structural features, but the sample preparation required can lead to significant changes in the materials morphology. EM requires many preparative steps to allow structures to be imaged including the application of an ultrathin coating of carbon, gold, or platinum. These are applied to conduct away accumulated surface electrostatic charge [30]. Furthermore, the requirement that TEM samples are prepared as thin films in order to allow electrons to be transmitted through the sample may require mechanical abrasion or ‘blotting’ of the sample to achieve this [14]. Supramolecular materials which are particularly sensitive to shear forces, will be particularly sensitive to morphological changes from the stresses of abrasive thinning. SEM and TEM also may require a fundamental change in the material in order to investigate it, as their high-vacuum environment necessitates that samples are dried. This is problematic for gels in particular, as gels are mostly solvent by composition, it is to be expected that the drying procedures required may lead to a change in morphology due to effectively concentrating the sample, leading to more aggregation, any salts present in the solution could also crystallise and disrupt the network, or change the stability and solubility of any charged structures present. All of these issues could result in statistics which are not representative of the structures in situ [31]. To overcome this, cryogenic techniques are widely used to allow the investigation of a material in its hydrated state.

Cryogenic techniques utilise a vitrification process to maximise the formation of vitreous ice to minimise the formation of ice crystals which can disrupt the material structure. The ability of cryo-EM to probe the structure of soft nanostructured materials has led to a number of reviews discussing its pivotal nature in supramolecular material characterisation [32]. One of the most common methods of vitrification involves plunging the hydrogel into a liquid propane/ethane mixture, transferred under vacuum to a preparation chamber where it is fractured, sublimed, and coated for imaging [14,33]. While propane/ethane is the most common cryogenic medium, various media exist with different heat transfer capabilities which can lead to differences in the vitrified structure [14,34]. It is important to consider that the increased thickness of cryo-SEM reduces the surface area-to-volume ratio which reduces the cooling rate reached in the vitrification process. High-pressure freezing (HPF) methods are capable of fixing samples 100 times thicker than plunge freezing. HPF reduces the growth of crystals during the ﬁxation of cryo-SEM samples by using very high pressures of liquid nitrogen, in the order of 210 MPa, and has been reported to improve the preservation of cryo-SEM specimen nanostructure [35–36]. While allowing thicker samples to be investigated avoids artifacts introduced by preparing thinner samples, cryo-SEM is limited to investigating the surface topology which is not necessarily the same as the bulk morphology.

**Figure 7:**
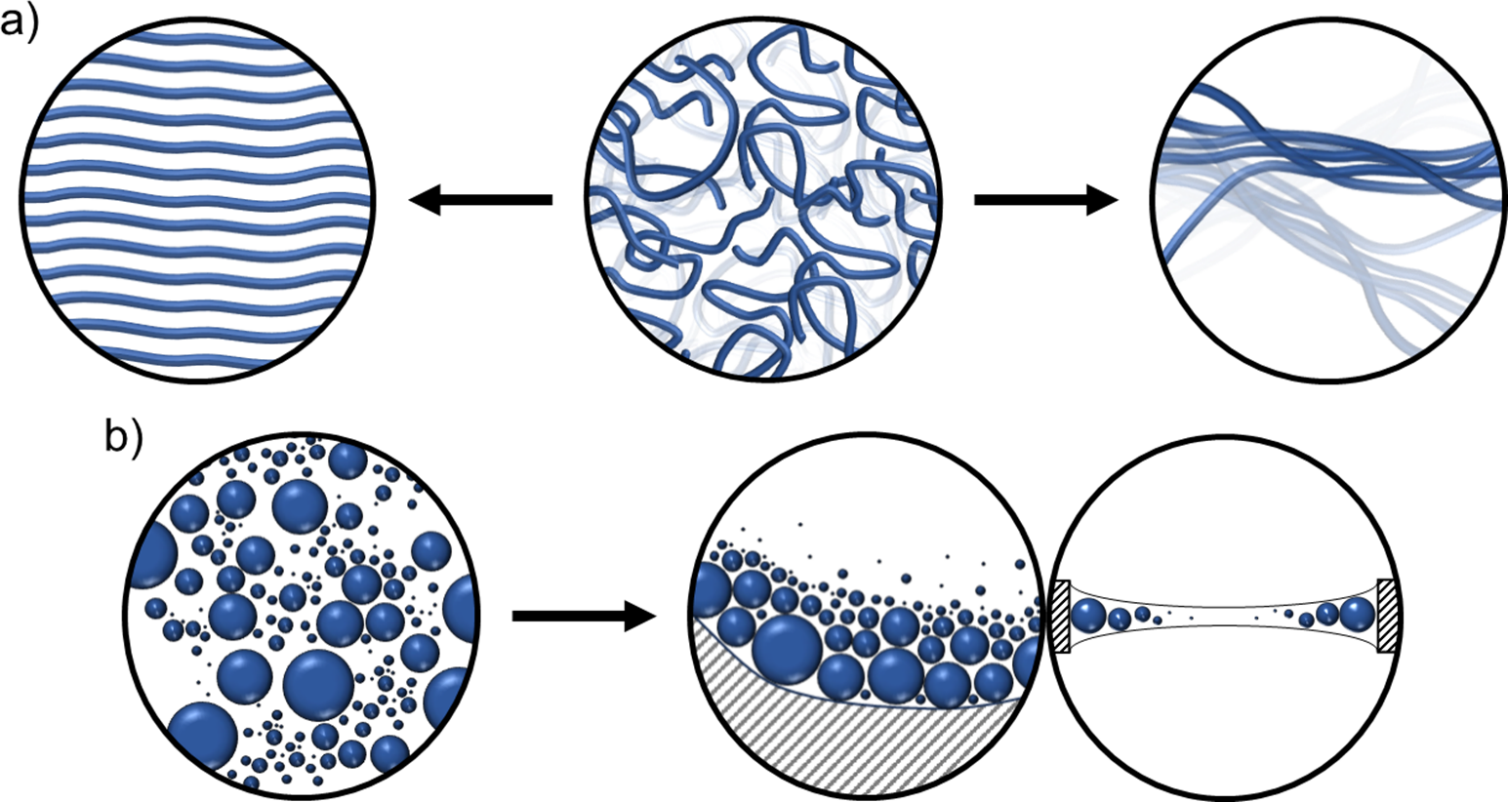
Cartoon of artifacts caused by blotting and thinning. a) Alignment of threadlike micelles (left) [32] and the transformation of threadlike structures into transient lamellar structures (right) [40]. b) Top-down (middle) and cross-section (right) views showing the segregation of structures with small assemblies pushed towards the thin central area and larger ones driven to the thicker areas at the cryo-TEM grid boundary [32].

Cryo-TEM can better probe three-dimensional structures, however, it is important to consider the additional artifacts that arise in cryo-TEM sample preparation. Cryo-TEM requires a thin film sample which can be difficult to prepare with a gel sample [37]. Blotting is a standard technique used to remove excess material from a TEM sample grid, however, even with the advent of robotic instruments it has been shown to be wrought with issues [38]. Not only are the preparations a nontrivial task, but the resulting films are often highly variable, non-uniform, and subject shear stresses which can align, segregate, or transform structures (Figure 7) [32,39–40].

Though assemblies at multiple length scales can be captured by cryo-TEM, blotting can cause larger structures to be expelled from the sample, limiting the ability of cryo-TEM to observe the range of hierarchical assemblies [32]. Another significant concern posed by blotting is the brief introduction of an air–water interface which can lead to preferential organisation of particles, as well as unpredictable changes in local concentration on the TEM grid [41]. To overcome such limitations, methods such as those by Arnold et al. have been developed to achieve the preparation of cryo-TEM grids without the need for blotting [42]. A recent review of cryo-TEM preparation techniques covers a number of deposition techniques, their benefits, and limitations was performed by Weissenberger et al. [34]. Even when successfully prepared, the limited thickness can make imaging gel networks difficult [31]. It should also be considered that changing the surface or vessel in which the material is prepared can lead to a change in the microstructure [43]. Therefore, the resulting material on the microscopy grid may not be representative of the typical structures even before drying or vitrification. It is also important to note that any electron microscopy image represents only a small portion of the overall sample and so the resulting characterisation may not account for inhomogeneities throughout the bulk network [2].

Another significant limitation posed by cryogenic microscopy techniques is their compatibility with organic solvent systems. Firstly, the vitrification procedure poses significant problems for the typical cryogens of liquid nitrogen and liquid ethane. Whilst liquid nitrogen is compatible with organic solvents, the lower cooling rate of ≈7000 K/s may be insufficient to vitrify the materials within organic solvents. Whereas the cooling rate of ethane of up to 100,000 K/s would overcome this, most organic solvents are soluble in liquid ethane making it incompatible with such systems [36,44]. Furthermore, even when vitrification is achieved, organic solvents are much less stable than aqueous samples under the electron beam. Currently, a typical high resolution cryo-TEM study generally requires a dose of ≥100 e^−^/Å^2^, however, vitrified organic solvents have reportedly shown beam damage effects at doses of <50 e^−^/Å^2^, making high-resolution cryo-TEM infeasible for many organic solvent systems [44].

**Figure 8:**
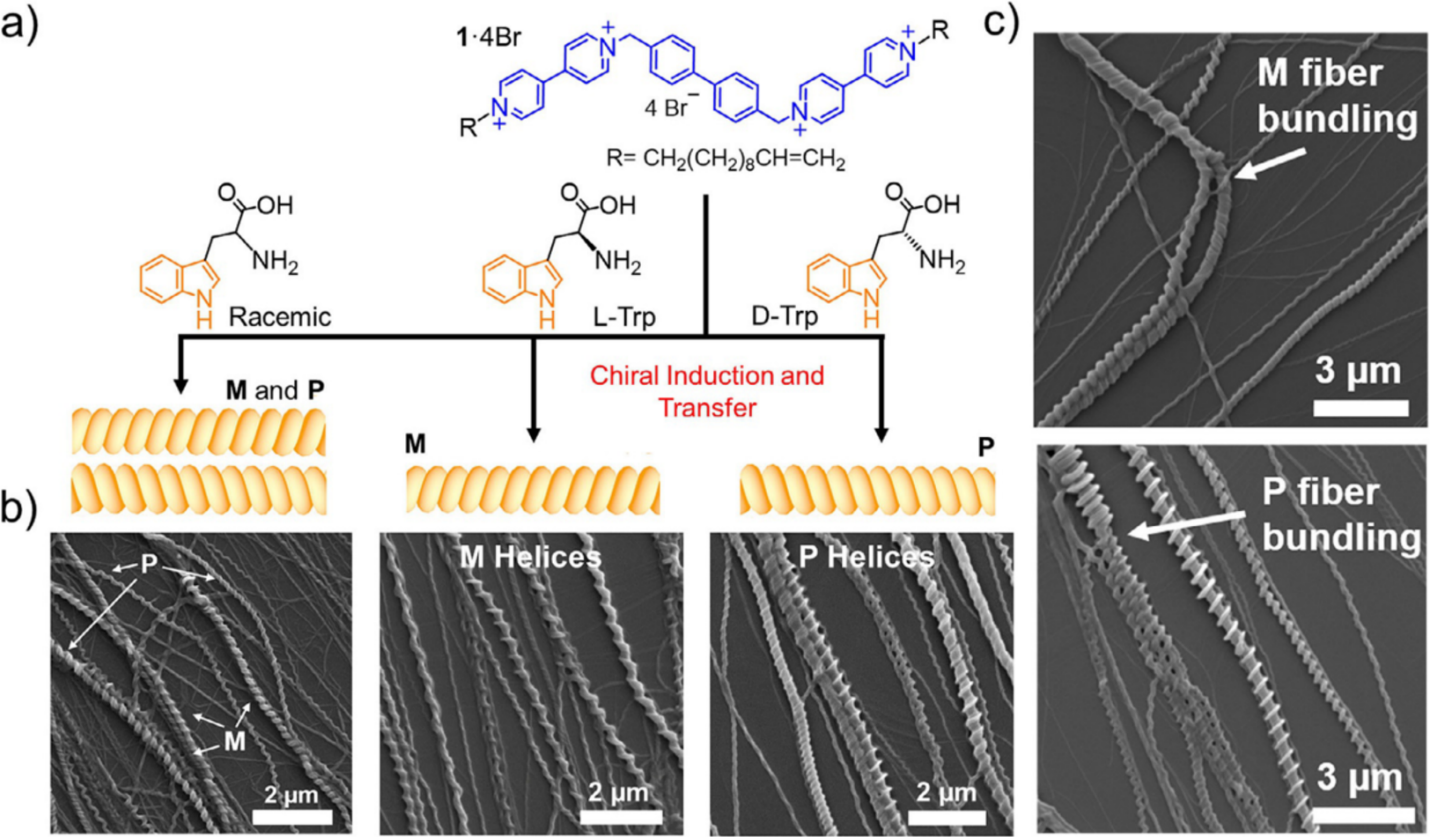
(a) Chemical structures of monomer compounds and a schematic of the resulting chiral helical structures. (b) SEM images of the resulting supramolecular helical fibres. (c) SEM images depicting fibre bundling leading to larger fibres with conserved helical chirality. Figure 8 was reproduced from [45], T. Yuan et al., “Assembly and Chiral Memory Effects of Dynamic Macroscopic Supramolecular Helices”, Chem. Eur. J., with permission from John Wiley and Sons. Copyright © 2018 Wiley-VCH Verlag GmbH & Co. KGaA, Weinheim. This content is not subject to CC BY 4.0.

Whilst imaging may have some caveats, it can be a hugely powerful tool when looking at larger areas of materials and looking for uniformity of samples. In materials formed from fibrous nanostructures, the interactions between fibres often dictate the mechanical properties of the material [44]. Therefore, it is crucial to be able to characterise the topology of these structures to better understand how they aggregate. Imaging techniques allow structural elements of a system to be directly observed. This is a significant advantage over scattering which does not provide a direct visualisation, and can be used to better understand the process which gives rise to the observed structure. This advantage was demonstrated by Yuan et al. with their investigation of supramolecular helices [45]. SEM was used to investigate the structures resulting from the self-assembly of a bis-bipyridinium-based compound (1·4Br) resulting in helical supramolecular fibres with different chirality induced depending on the enantiomer of tryptophan present. The direct images obtained from SEM allowed the chirality of the resulting fibres to be easily observed, indicating that the hydrogel matrix induced by racemic tryptophan was comprised of racemic amounts of left-handed (*M*) and right-handed (*P*) helices (Figure 8) [45]. Due to the orientational averaging of scattering techniques, directional information is lost. Subsequently, extracting information such as chirality cannot be achieved without additional information such as that obtained from direct imaging [46]. A more comprehensive discussion of the capabilities and limitations of scattering techniques is included later in this review.

**Figure 9:**
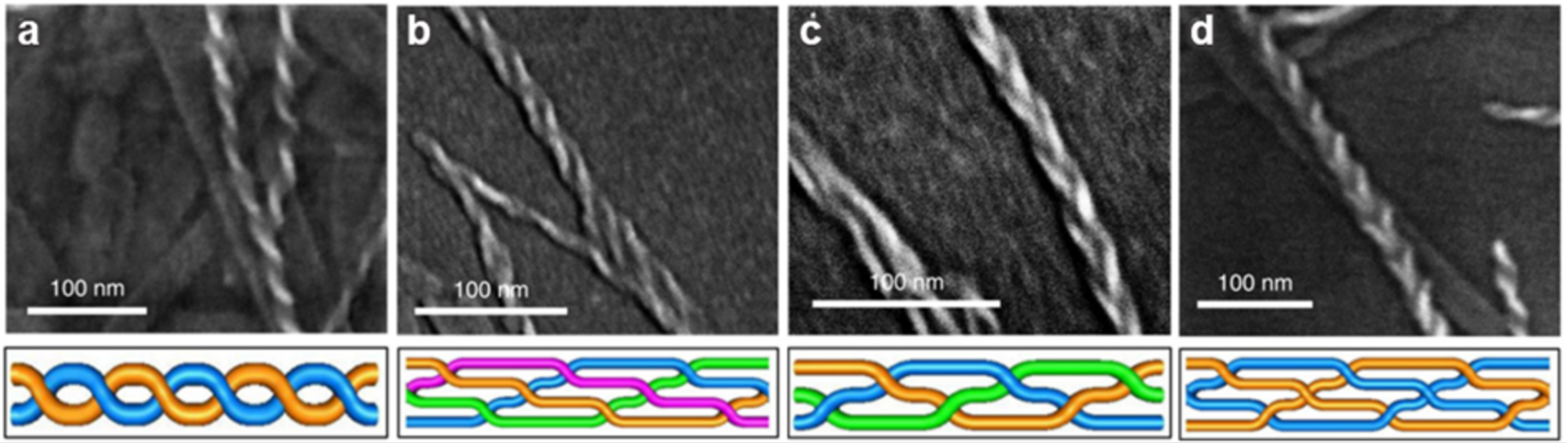
Commonly observed entanglements of urea-based supramolecular helices. (a) Double helix, (b) quadruple helix, (c) Brunnian braid, and (d) nested helices. Figure 9 is from [44] and was adapted by permission from Springer Nature from the journal Nature Chemistry (“Braiding, branching and chiral amplification of nanofibres in supramolecular gels“ by C. D. Jones; H. T. D. Simmons; K. E. Horner; K. Liu; R. L. Thompson; J. W. Steed), Copyright 2019, The Author(s), under exclusive licence to Springer Nature Limited. This content is not subject to CC BY 4.0.

SEM was also able to identify fibre–fibre bundling where smaller fibres further assemble to form thicker fibres while maintaining their helicity. This allowed for the formation and observation of enantiomerically pure fibres of increasing size (Figure 8c). The direct image yielded by microscopy allows the self-assembly process to be easily interpreted, where the rationalisation of such structures may not be achievable with complex indirect imaging techniques. This advantage is epitomised by Jones et al. whose self-assembling urea compounds form helices which can further entangle into a number of significantly more complex braided structures observable by SEM (Figure 9) [44].

An important feature of self-assembled networks is the defects that arise in the fibre structures as shown in the characterisation of β-hairpin peptide hydrogels by Yucel et al*.* [47]. The cryo-TEM characterisation indicated that the formation of the hydrogel was directed by self-assembly defects giving rise to branches in the fibrillar structure which was distinct from other reported biopolymeric systems. If the cross-link was a result of entanglement as with similar systems, then the junction would show an increase in contrast due to the two fibril cross-sections increasing the interactions with the electron. The uniform contrast was instead indicative of the branching as a result of valine facial collapse of β-hairpin, giving rise to multifunctional branches which allow cross-linking.

**Figure 10:**
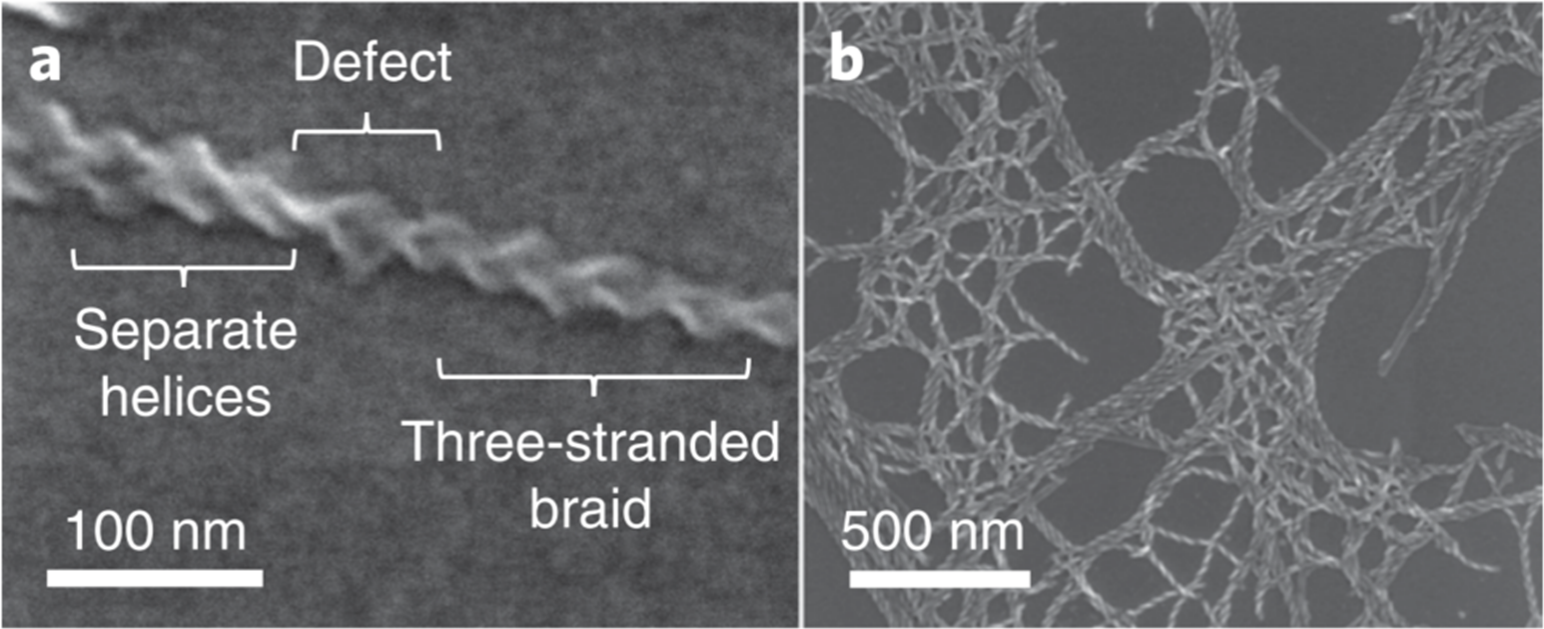
(a) SEM image of a single three-stranded braid showing a defect in which the braid separates into separate fibrils. (b) SEM image showing that the separation of branch points in dried gel samples is highly variable. Figure 10 is from [44] and was reprinted by permission from Springer Nature from the journal Nature Chemistry (“Braiding, branching and chiral amplification of nanofibres in supramolecular gels“ by C. D. Jones; H. T. D. Simmons; K. E. Horner; K. Liu; R. L. Thompson; J. W. Steed), Copyright 2019, The Author(s), under exclusive licence to Springer Nature Limited. This content is not subject to CC BY 4.0.

The capabilities of imaging in elucidating the mechanism of aggregation is further exemplified by Jones et al. [44]. It was shown that SEM could directly observe structural defects, resulting in the collapse of helically braided structures into their constituent fibrils which can interact with other fibres (Figure 10). The branching density resulting from these defects could be gauged by SEM since the braid crossings are topologically fixed and act as permanent junctions [44]. Cross-linking caused by such defects can only be observed by localised techniques, since they do not represent the bulk averaged structure. Investigating such local structures can be achieved with imaging techniques while the statistical-averaging of scattering models results in the loss of this information.

**Figure 11:**
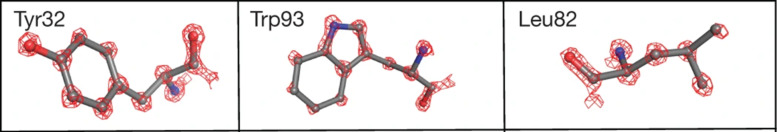
Visualization of individual atoms at 1.25 Å resolution. Three apoferritin residues are shown at high density threshold. The true atomic resolution of the map is shown by the clear separation of individual C, N, and O atoms at high thresholds. Figure 11 is from [48] and was adapted by permission from Springer Nature from the journal Nature (“Atomic-resolution protein structure determination by cryo-EM“ by K. Yip; N. Fischer; E. Pakni; A. Chari; H. Stark), Copyright 2020, The Author(s), under exclusive licence to Springer Nature Limited. This content is not subject to CC BY 4.0.

The staggering advancement of the field of electron microscopy continuously provides new opportunities to characterise structures with increasing resolution. Recently, Yip et al. was able to significantly improve upon the state of the art in cryo-TEM to achieve unprecedented structural detail. With a structural resolution of 1.25 Å, individual atoms and hydrogen density in the apoferritin can be visualised (Figure 11) [48].

We highlight here two reviews which discuss the fast-paced development of cryo-TEM imaging. Cheng et al*.* has reviewed the decades of cryo-TEM developments which have given rise to single-atom resolution techniques [49]. Chua et al. discussed a number of factors including advancements in sample preparation, instrumentation, and processing which have improved rapidly [50]. These factors together have allowed electron microscopy to become better, faster, and cheaper; establishing it as a dominant technique for structural characterisation.

### Scattering techniques

Small angle scattering techniques have been used to investigate soft matter systems for decades, with pioneering work such as that by Pierre Térech showing how such techniques can be applied to probe both local and long-range structures of 3D networks [51]. In the decades since there has been a wealth of reviews on soft matter characterisation as interest in such systems increases, and techniques develop at a rapid pace [10–11,52–53]. SAS techniques include small angle X-ray scattering (SAXS), small angle neutron scattering (SANS), and their wide angle (WAXS) and ultra-small angle (USAXS and USANS, respectively) analogues have various advantages and drawbacks compared to one another (Table 2), as well as microscopy techniques in the characterisation of soft matter self-assemblies.

**Table 2:** Comparison of scattering techniques [54–59].

	WAXS	SAXS	USAXS	SANS	USANS

length scales (nm)	0.1–1	1–100	20–3000	2–200	500–2000
sample volume	0.1–500 µL			50–3500 µL	
radiation sources	lab and large facility			large facility only	
typical large facility acquisition time	seconds			minutes	
solvent	deuteration not required for contrast			deuteration often required for contrast	
artifacts	beam damage			isotope effects	
advantages	time-resolved analysis, high-throughput			contrast matching, magnetic scattering	

**Figure 12:**
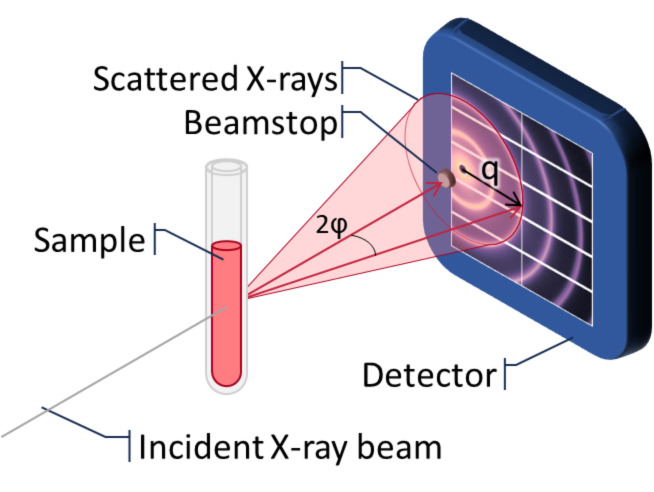
Cartoon of a general small-angle scattering setup.

These techniques fire a monochromatic beam of radiation through a sample, with the majority of the radiation being transmitted (and blocked by a beamstop) without interacting with any structures in the sample. A small amount of the radiation will interact with either the electron density (SAXS) or the nuclei (SANS) of the structures in the sample resulting in Thompson scattering that can be detected. The resulting scattering is characterised by the scattering vector (**q**) which is the result of a photon or neutron of wavelength (λ) scattering off the sample at an angle of 2φ (Equation 1 and Figure 12).


[1] $$q=\frac{4\pi \sin\varphi }{\lambda }$$

Typically, a 2D detector measures the scattered radiation to determine the scattering intensity (I(**q**)) and is measured as a function of **q**. The scattering vector has units of reciprocal space, so the smaller low-q measurements represent larger real-space distances (*d*) of repeating structures (Equation 2).


[2] $$d=\frac{2\pi }{q}$$

**Figure 13:**
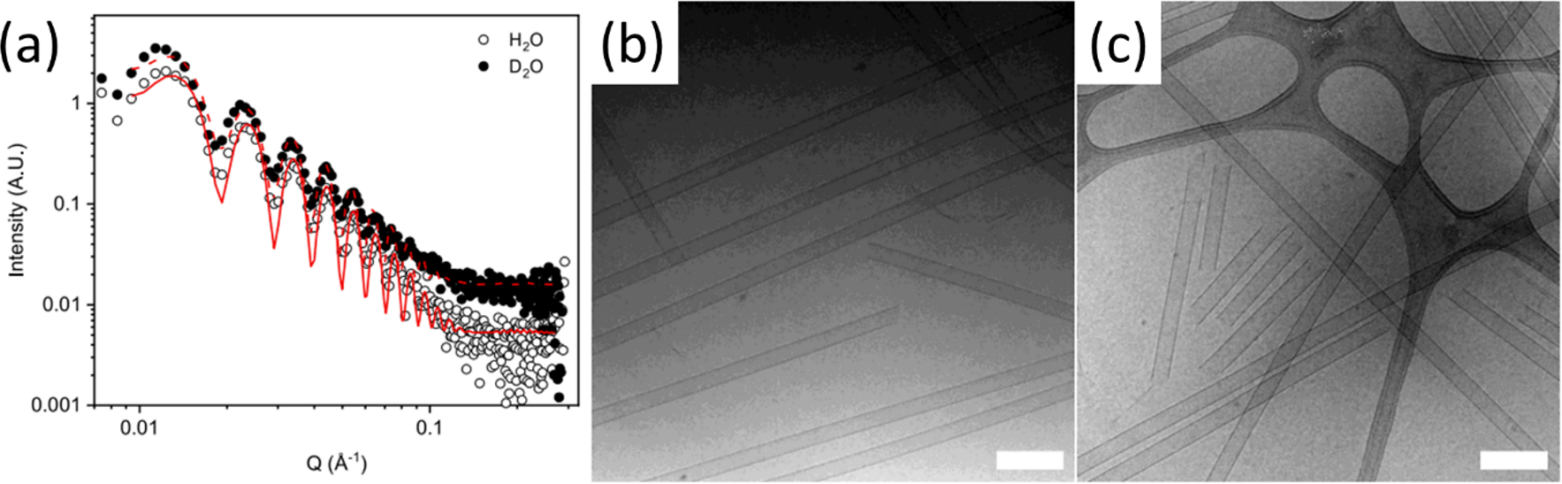
(a) SAXS data and fits for solution in H_2_O (open symbols) and D_2_O (closed symbols). Cryo-TEM data for solution in (b) H_2_O and (c) D_2_O. Figure 13 was adapted from [58], K. McAulay et al., “Isotopic Control over Self-Assembly in Supramolecular Gels”, Langmuir, © 2020 American Chemical Society, distributed under the ACS AuthorChoice/Editors’ Choice via Creative Commons CC-BY Usage Agreement, https://pubs.acs.org/page/policy/authorchoice_ccby_termsofuse.html. This content is not subject to CC BY 4.0.

The scattering pattern characterises the electron or nuclei density throughout the sample, which is directly affected by the arrangements of molecules and atoms throughout the structure. Therefore, the observed scattering carries bulk-averaged information that is a statistical average of the structures across the macroscopic irradiated volume [10]. Depending on the q-range measured, different structural scales can be investigated. As q increases, smaller structures are probed allowing the topology to be determined. For assembled structures in a solvent, there needs to be an appropriate difference in scattering length density (SLD) of the material compared to the solvent (called the contrast). There are online calculators that can be used to calculate the SLD of molecules and solvents [60]. This is so that the solvent can be suitably removed from the scattering pattern and a model then be used to fit only the material of interest. For X-ray scattering, most solvents have a suitable difference in SLD and so can be used, but should be checked. However, in neutron scattering the protons scatter very strongly, and therefore deuterated solvents are used to essentially hide the solvent to ensure the solvent does not contribute to the scattering pattern and contrast can be achieved this way. Another way to create this contrast is to selectively deuterate the molecule of interest and leave the solvent deuterated. This gives the advantage of contrast matching, where parts of the molecule can be hidden so temporal mapping can be achieved, but all add to the cost and complexity of the experiments. This is discussed in more detail further on. A number of models exist which describe the expected scattering that would arise from a wide range of structural topologies. This fitting allows multiple parameters to be determined. For example, self-assembled materials form worm-like micelles which can be described by various cylindrical models parameters including structure length, Kuhn length, structure radius, and axis ratio [61]. Scattering is representative of the overall system in comparison to the snapshots of individual micelles observed in microscopy techniques [37]. One of the foremost benefits of SAS is the capability to perform in situ measurements, wherein samples can be analysed with minimal change to the materials environment. This allows for structures that are more representative of their typical environment. To achieve this, many specialised cells have been developed to control various environmental parameters including temperature, pressure, irradiation, electromagnetic fields, and flow [62]. Sample environments can also be as simple as a capillary at ambient conditions. Without a need for the sample environment to be under vacuum, as is required for some microscopy techniques as previously discussed, SAS allows for volatile solvent systems to be characterised. In addition, cells that allow for the coupling of SAS with non-X-ray techniques continue to be developed; these include spectroscopy, rheology, and electrochemistry [62–64]. Ascribing changes in properties of interest directly to changes in the structure further elucidates the interesting properties of self-assembled materials, and better enables rational design. It is important, however, to consider that small changes in the sample environment can lead to significant changes in the self-assembled structures. For example, in aqueous samples, the use of D_2_O is necessary to produce contrast in SANS analysis and may be assumed to provide an in situ environment. While D_2_O is chemically identical to H_2_O, it does exhibit differences in properties including density, viscosity, hydrogen-bond strength, a more pronounced hydrophobic effect. As hydrogen bonding and hydrophobicity are critical to hydrogel characteristics it has been shown that substituting the hydrogel solvent with D_2_O can lead to structural changes in self-assembled aggregates (Figure 13) [58]. Therefore, by allowing for a better understanding of how self-assembled systems are structured, SAXS and SANS can be a more accurate tool for identifying structural characteristics that give material properties of interest. However, care must be taken to sure a truly representative environment is probed.

**Figure 14:**
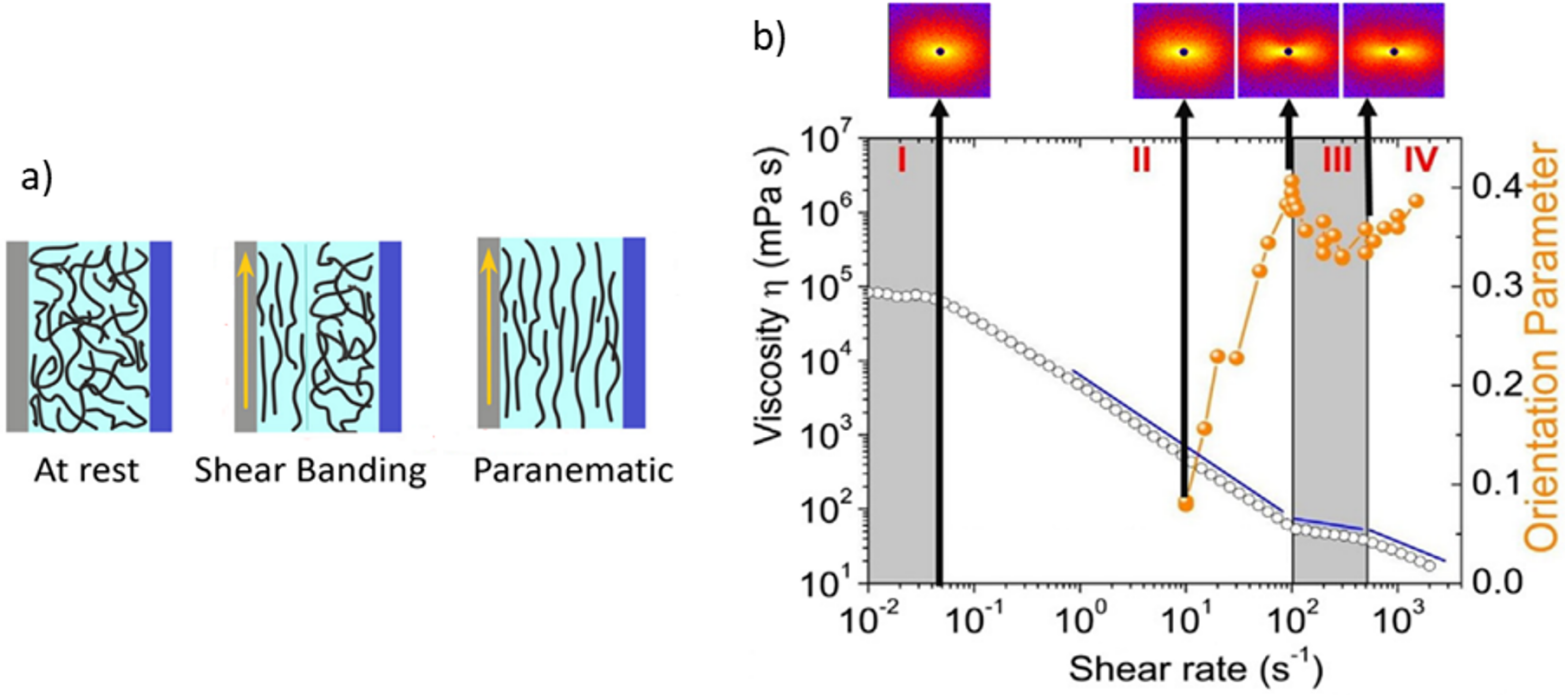
(a) A cartoon illustrating the orientation phases caused by shear alignment of WLMs. (b) Rheological profile with different orientation phases. I: Weakly oriented micelles; II: shear banding region; III: paranematic phase; IV: shear-thinning region of unexplained origin. Above are representative 2D-SANS scattering patterns which were used to define the orientation parameter of WLMs. Figure 14 was adapted from [67], Journal of Colloid and Interface Science, vol. 560, B. Arenas-Gómez; C. Garza; Y. Liu; R. Castillo, “Alignment of worm-like micelles at intermediate and high shear rates”, pages 618-625, Copyright (2019) with permission from Elsevier. This content is not subject to CC BY 4.0.

Coupling rheology with SAS (rheo-SAS) is of particular interest for soft matter applications due to its ability to identify and rationalise how changes in self-assembled structures affect the mechanical behaviour in bulk networks [65–66]. An investigation of the shear alignment behaviour of worm-like micelles (WLMs) in solution by Arenas-Gómez et al. highlights how rheo-SAS can allow for the interpretation and understanding of material behaviour which rheology alone cannot provide. By using rheo-SANS, the structural order of the micelle solutions could be determined under flow. Worm-like micelles can align under shear, however, it was shown that under sufficiently high strain a plateau in viscosity is reached before again undergoing shear-thinning under increasing shear [67]. This plateau had been observed in various systems, but an origin of the phenomenon had not been described. However, the unexpected behaviour could be rationalised as friction effects by quantifying the degree of orientation, as determined from anisotropic 2D-SANS scattering patterns (Figure 14) [67]. The dynamic nature of such rheological measurements can only be achieved in situ. Imaging techniques such as TEM and cryo-TEM are incompatible with rheology due to the necessity of freeze drying or vitrification during sample preparation.

**Figure 15:**
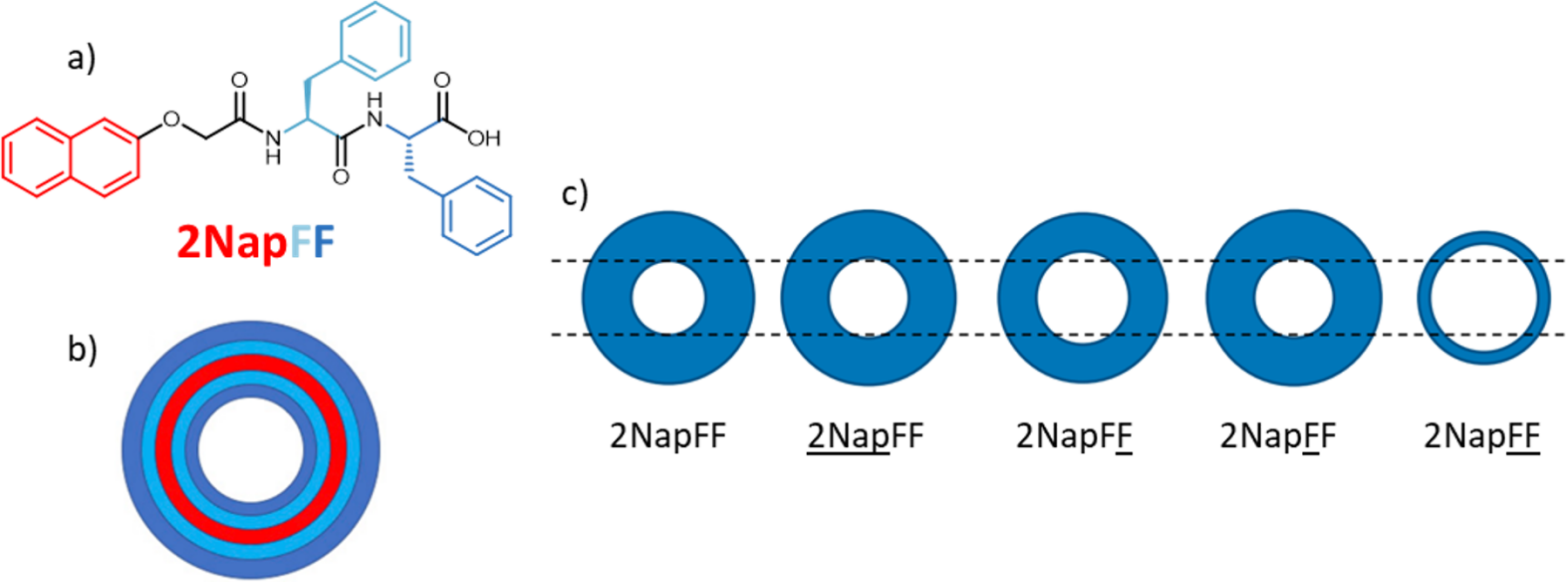
(a) Chemical structure of 2NapFF and (b) a cartoon cross-section of the hollow cylinder structure formed by 2NapFF in D_2_O with colour coding for the different sections. (c) Cross-section of hollow cylinder structures formed for selectively deuterated forms of 2NapFF in D_2_O, with sizes scaled to those derived from SANS fits. An underline has been used to denote the selectively deuterated sections of 2NapFF. Figure 15 was adapted from [68] (© 2020 E. Draper et al., published by Elsevier, distributed under the terms of the Creative Commons Attribution 4.0 International License, https://creativecommons.org/licenses/by/4.0).

As mentioned above, SANS has the additional benefit of contrast matching where the scattering of certain compounds or aggregates can be screened out to better identify and quantify different nanostructures which result from self-assembly [68]. In addition, contrast matching can investigate how multiple components either self-organise or co-assemble [69–70]. We have previously used the power of contrast matching to investigate self-assembly by selectively deuterating 2NapFF to identify the molecular packing structure in D_2_O solutions (Figure 15) [68]. SANS allowed the surfactant-like aggregation of 2NapFF to be elucidated which provides the opportunity for the directed tailoring of the nanostructure topology. To further characterise the structures of the self-assembled hollow cylinders, additional investigations are required. We discuss later how these structures can be further elucidated by using scattering and microscopy in tandem.

SAS is also a powerful technique since measurements are performed in situ. Measuring in situ avoids drying effects which are a limitation of analogous microscopy measurements [37]. Furthermore, by investigating samples in situ, SAS techniques can follow a change in structure over time upon an initial stimulus such as a change in temperature. Jamieson et al*.* were able to utilise in situ SANS to characterise the ageing effects of gels formed upon the cooling of hot pyromellitamide solutions [71]. By comparing the fit of the full model to the low-q region only a higher level of hierarchical self-assembly, previously unobserved by microscopy, was characterised. The fit of the full model identified the one-dimensional fibres formed by the stacking of molecules, while the low-q fit identified a larger clustering feature which could be described by the formation of asymmetric multifibre braided clusters. SANS showed that as the gels continue to age, the radii, persistence length, and contour length continue to increase. This revealed that the morphology of the self-assembled gels can undergo significant structural change over time, highlighting the dynamic nature of supramolecular materials and showing how an understanding of the kinetic processes probed by such in situ investigations are essential to understanding the relationship between gel structure and their bulk properties [71].

As discussed previously, scattering provides bulk-averaged information that is a statistical average of the structures across the macroscopic irradiated volume [10]. This makes the meaningful application of SAS techniques to heterogeneous systems challenging [72]. As the topological complexity of the system increases, so too does the difficulty of modelling the data. This can result in the number of modelling parameters becoming excessive, resulting in overfit data that becomes increasingly meaningless. While often there is a focus on designing processes to maximise the uniformity of supramolecular materials [73], there is increasing interest in the design of inhomogeneous systems [74–75]. Such materials include the rapidly advancing field of gradient hydrogels which possess a gradual or abrupt change in properties throughout the body of the hydrogel. Scanning-SAXS reduces the size of the X-ray beam such that a nanobeam and microbeams to probe local structures in an area on the order of the beam radius [72]. This allows the structure of small sample areas to be investigated, however, the resolution is restricted by the limited focus of X-ray beamline, which has reached the sub-100 nm [76]. The limited resolution afforded by scanning-SAXS means structures can be probed from the micron scale down to the tens on nanometres [77–78]. This makes in situ measurement of the complete nanoscale topology inaccessible for inhomogeneous self-assemblies. Despite the limitations, accessing statistical in situ measurement of the microstructure could be crucial to characterising gradient systems. It is important to consider that the container in which a gel is prepared can impose morphological changes in the sample [43], and so this issue extends to the preparation of gradient hydrogels for in situ measurements. The different preparation environments required for different techniques would therefore make it difficult to correlate the gradient of structures observed in complementary techniques. It is noted in a review by Zinkovska et al. that there is a lack of literature discussing the characterisation of such systems. Characterisation of these systems is typically achieved with microscopy techniques [74]. We believe that scanning-SAS techniques could be a powerful complement to the characterisation techniques used to develop our understanding the microstructure of these materials. It should be noted that microbeam SAXS setups are not ubiquitous at synchrotron facilities and can be limited to strong scatterers so the application and accessibility of such techniques can be limited [77].

It has been previously established that there is a direct correlation between the bulk properties of self-assembled materials and their nanostructures [79–80] and microstructures [81–82]. Recently, Adams et al. utilised SANS to quantify the fibrillar structures formed by the self-assembly of amino acid-appended perylene bisimides (PBIs) [83]. They found that the average fibre length was a critical nanostructural feature for affording a desirable mechanoresponsive property, that is the change in conductivity under a bending force, in PBI films. Therefore, a potential mechanism could be identified which allows for the directed design of compounds which form mechanoresponsive aggregates, by indicating that a focus on increased fibre length is required. Information at longer length scales may be lost due to the limited q range provided by SANS experiments. This indicates that SAS techniques, while better able to quantify structures in a robust statistical manner, are often fundamentally limited to describing cross sectional features of supramolecular fibres. Given that characterising the entirety of the supramolecular network is required to truly understand it SAS techniques cannot be solely relied upon to obtain a complete understanding of supramolecular materials. Therefore, combining the knowledge obtained from SANS investigations in conjunction with microscopy methods, as discussed below, enables investigations over length scales and could prove effective at elucidating design principles for such a desirable class of materials.

### Combining techniques

In the previous sections, we have discussed the merits and limitations of numerous microscopy and scattering techniques as summarised in Table 3. This table highlights the complementary nature of these techniques and how, in conjunction with one another, they can overcome the shortfalls each exhibit on their own.

**Table 3:** Comparison of microscopy and scattering techniques with advantageous traits highlighted in bold.

	microscopy	scattering

local structure and detail	**easily detected**	details lost
average structure	difficult to obtain	**always obtained**
preparation artefacts	difficult to prevent	**none**: measurement in-situ
resulting data	**direct pictures** with poor statistics	difficult to interpret, **good statistics**

**Figure 16:**
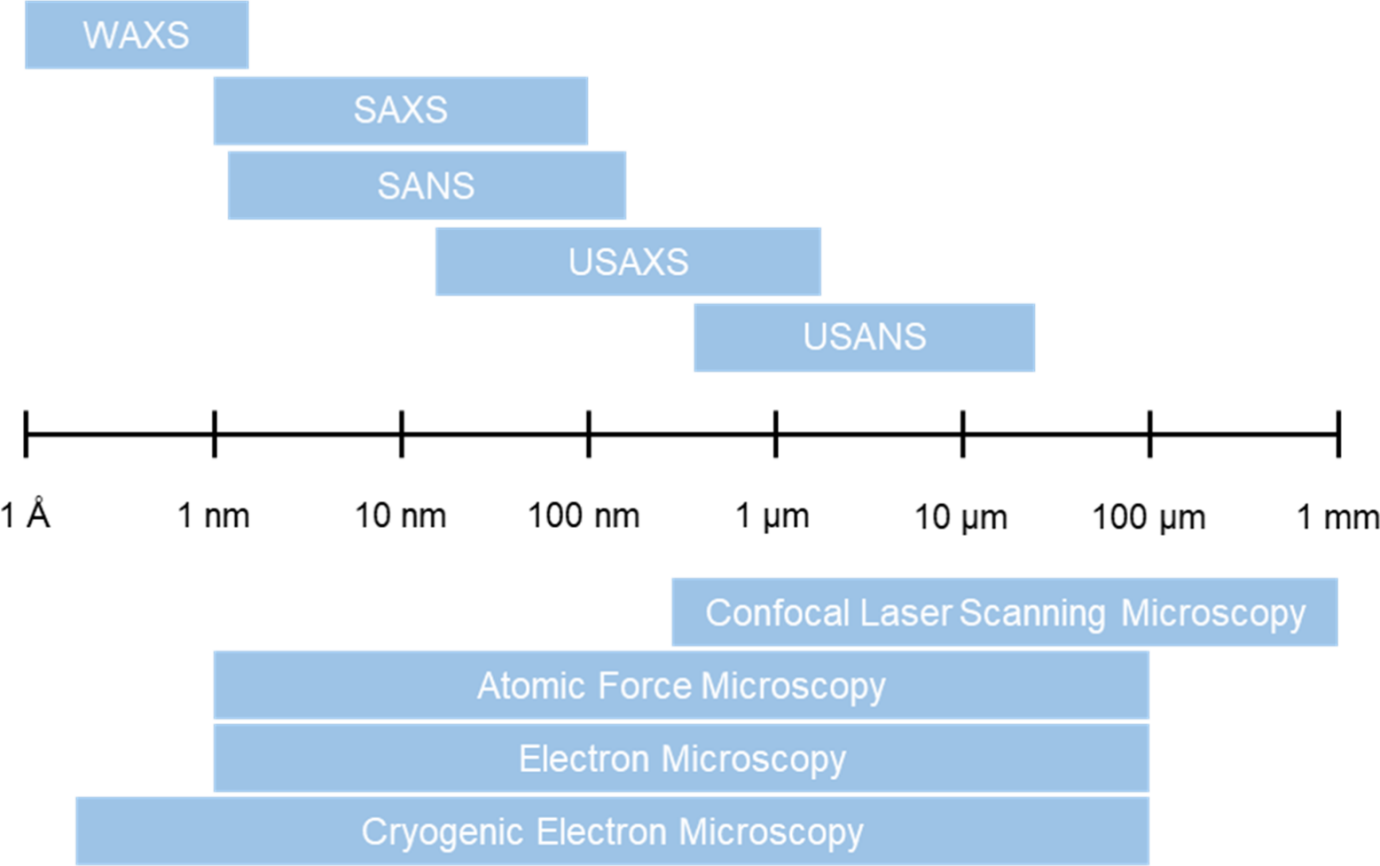
Length scales of scattering and imaging techniques [16,54–55].

In addition to the characteristics described in Table 3, it is important to consider the length scales of interest. As previously explained, the properties of supramolecular materials arise due to the assembly across many length scales (Figure 1), and as such characterisation is required across multiple length scales to fully understand these systems. The scale disparity between the diameter of the fibres at the nanoscale, and that of the microscale structure spans 3 to 4 orders of magnitude. No single microscopic technique can accurately characterise both scales, and so a combination of techniques is required [12]. Similarly, scattering requires a combination of wide angle (WAXS), small angle (SAXS/SANS), and ultra-small angle scattering (USAXS/USANS) to characterise structures over a range of magnitudes [54]. However, scattering can only probe lengths up to the tens of microns with USANS, whilst EM techniques are capable reaching hundreds of microns in scale and CLSM even further into the millimetre range (Figure 16). It has been shown by Ornatska et al. that supramolecular assemblies can give rise to microfibres with lengths in the order of tens of micrometres, all the way to centimetre scale assemblies [84].

Overcoming such restraints is possible by using several techniques in tandem to probe the entire length scale. Martin et al. previously investigated the hierarchical self-assembly across length scales using a combination of SANS and cryo-TEM, as well as other characterisation techniques. To investigate how the change in capping group changed the self-assembly of diphenylalanine, indole-FF, and carbazole-FF (F = phenylalanine) were characterised by SAXS. While both indole-FF and carbazole-FF were shown to have fibre radii that were quantifiable by SAXS, the model fit to the indole-FF showed the length to exceed the q range measured. Cryo-TEM was used to corroborate the presence of such fibres, and though they cannot prove the modelled length’s accuracy, they were able to show that the self-assembly occurred on these length scales [85]. This data was used to show that hydrophobicity was a key factor in controlling the self-assembly of these compounds.

**Figure 17:**
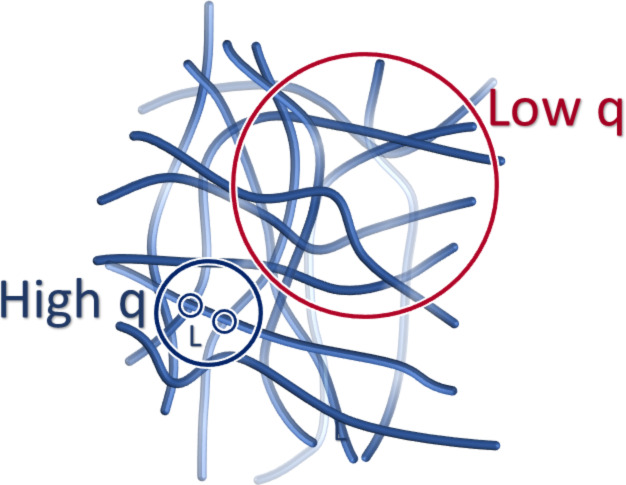
A schematic of a hydrogel network showing the significance of various parameters extracted from SANS. Red and blue circles represent scattering at distinct length scales. Red circle represents network morphology over hundreds of nanometres, characterised by low q scattering. Blue circle represents nanostructure over a few nanometres, characterised by high q scattering. Interfibrillar crosslink distances are represented by correlation length (L). Fibrillar density for network and nanostructure are represented by the low q and high q scattering. Figure 17 was redrawn from [79].

The work by Greenfield et al. shows the advantageous tuneability of peptide amphiphiles and their ability to form gels with significantly different properties depending upon the gelation trigger [86]. It was shown that the different entanglements and cross-links formed with the addition of different triggers were instrumental in controlling the rheological properties of self-assembled materials. Understanding such changes in structure is key to tailoring their mechanics [86]. A current limitation to understanding self-assembled materials is the difficulty in determining cross-linking throughout the network. Microscopy techniques are often used to probe the network, however, the significant drying artefacts can alter the network, and both SAXS and SANS do not probe the appropriate length scales to determine their prevalence, although some information can be obtained [87]. An investigation by Hule et al. used SANS, USANS, and cryo-TEM to correlate the stiffness of a hydrogel with the network morphology and nanostructure [79]. To investigate the effect of the gelator concentration on the density of the hydrogel network, SANS was used to determine the correlation length (Figure 17). As the concentration of the gelator increased, a decrease in the correlation length was measured which evidences a decrease in the distances between cross-links, and the pore sizes between fibrils. This corroborated direct evidence of the increasing number and cross-links observed by cryo-TEM. Separate rheological investigations of the system were used to understand the concentration dependence on the bulk rheological of the resulting hydrogel, and showed that the change in stiffness was directly related to the increase in the network density and the degree of cross-links with concentration [79].

While the direct images afforded by imaging techniques can be instrumental in identifying fibril morphology, preparation artefacts can hinder the quantification of structural features of the larger network by changing the morphology resulting in statistics which are not representative of the structures in situ [31]. Mears et al. have shown that when the fibres are hydrophobic and not heavily charged, microscopy techniques which require drying are not suitable for observing the primary fibres, and instead, are only capable of detecting larger aggregates of primary fibres [37]. The comparative investigations of SEM, cryo-TEM and SANS were critical in identifying this limitation of SEM. Cryo-TEM and SANS techniques are capable of probing the primary fibres, however, the discrepancy between cryo-TEM and SEM indicates that SEM was not representative of the fibrous network. The observation of fibres associating in the cryo-TEM as well as the detection of larger aggregates in SEM shows that there also existed some higher-order aggregates that were not detectable by SANS. Therefore, together scattering and microscopy techniques could better describe the hierarchical assembly of such supramolecular materials.

In addition to complementing length scales, microscopy can be used to probe materials that, at increasing concentrations, are incompatible with scattering techniques. Conversely, scattering can probe the structure in situ which leads to a more reliable characterisation of the structures since drying effects can lead to a change in morphology [37]. The combination of microscopy and scattering techniques to overcome these limitations was achieved by Firipis et al. where they used SAXS to quantify hydrogels formed from self-assembling bioactive peptides Fmoc-DIKVAV and Fmoc-FRGDF (D = aspartic acid, I = isoleucine, K = lysine, V = valine, A = alanine, F = phenylalanine, R = arginine, G = glycine) [88]. The physical characteristics of such a hydrogel can be tuned with the addition of the polysaccharide agarose. TEM showed that as the concentration of agarose was increased, Fmoc-FRGDF fibres remained stable. However, TEM showed Fmoc-DIKVAV fibre width increased as the concentration of agarose increased. SAXS was used to characterise the materials in 0.05, 0.1, and 0.2% agarose, however, due to the significant scattering afforded by agarose dominating the scattering curve, the diameter of the self-assembled fibres in higher concentrations of agarose could not be quantified. At the quantifiable concentrations, SAXS showed the diameter of fibres for both Fmoc-DIKVAV and Fmoc-FRGDF to be constant. The consistency of the structures was in contrast to the TEM results, but the in situ nature of the SAXS measurements made them a more reliable measure of the structures present. This showed that there was no correlation between the fibre width and the changing mechanical properties observed in the materials. The networks are peptide-dominated at low agarose concentrations, and agarose-dominated at high agarose concentrations, resulting in distinct changes in structural morphology. Instead the variation in mechanical properties were shown to arise from changes in the nanopore size (representative of the degree of entanglement in the self-assembled structure) as quantified by SAXS, and the microtopography as quantified by cryo-SEM [88].

Above we have provided a rationale for using a combination of microscopy and scattering techniques to characterise self-assembled systems, highlighting the benefits with a number of exemplary investigations. There are several papers that show such techniques corroborating one another, although they only make up a small number of such investigations. We caution that relying on a single method of characterisation may not provide a complete description of a material. Even when using more than one technique certain considerations should be made. Below we discuss these further.

### A priori knowledge

**Figure 18:**
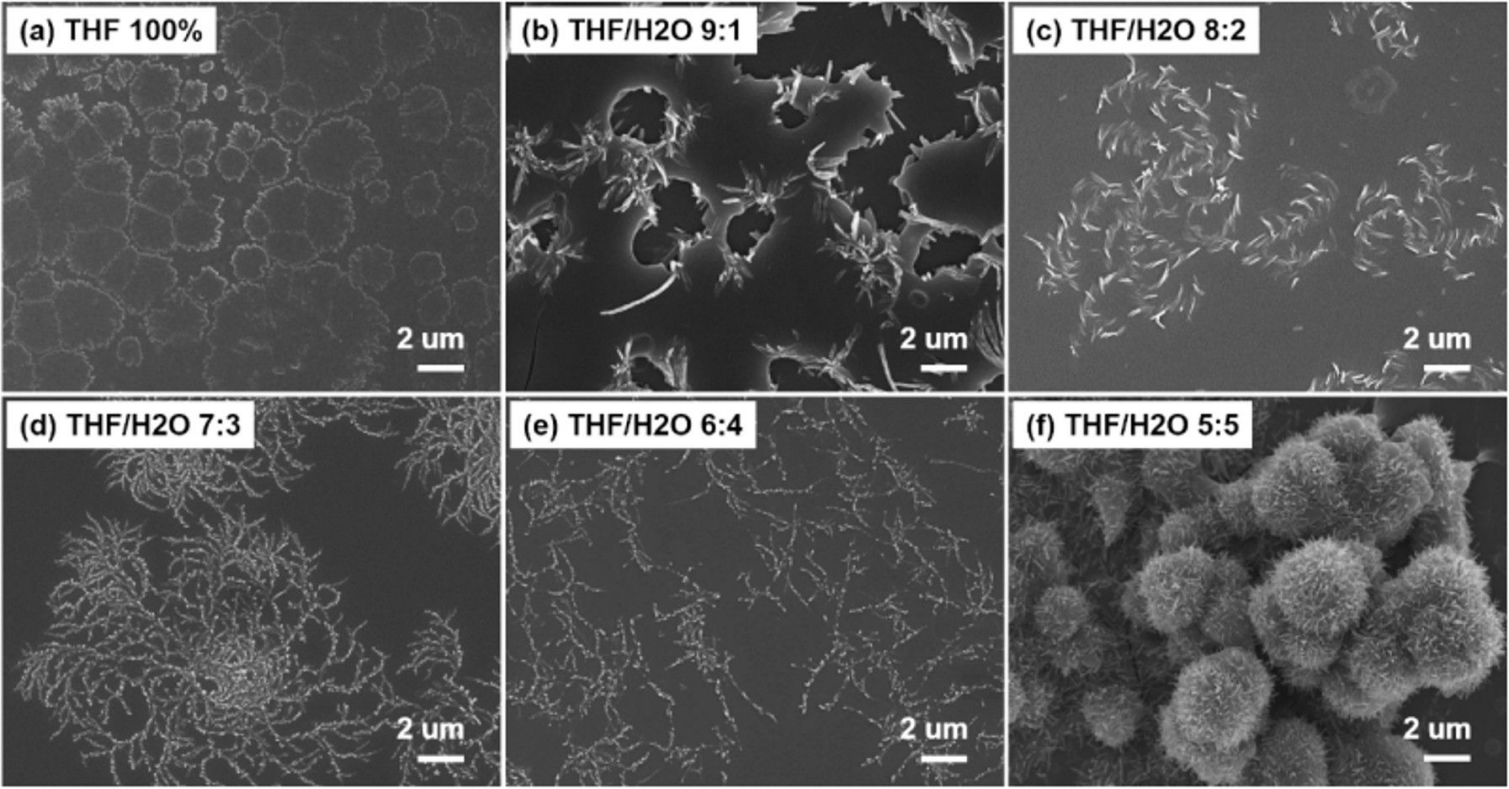
The morphologies of a co-assembled complex dependent on the solvent composition. Figure 18 is from [89] and was reprinted by permission from Springer Nature from the journal Nature (“Spiral fractal patterns via hierarchical assembly“ by L. Zhang; M. Deng; Y. Duan; X. Wen; Y. Jiang; H. Jiang; Y. Ma; M. Liu), Copyright 2021, Tsinghua University Press and Springer-Verlag GmbH Germany, part of Springer Nature. This content is not subject to CC BY 4.0.

While both microscopy and scattering techniques have proven to be pivotal in our understanding of self-assembled materials, it is important that the data obtained from such techniques cannot be used in isolation. For example, a wide variety of models are available to characterise scattering data, however, solving the equations leads to multiple solutions. With scattering, the ‘best’ model is often chosen from the fit that gives the lowest fit value (χ^2^); a fibrous system would be expected to fit to a type of cylindrical model rather than a Pringle model for example. Effectively applying such models requires a priori knowledge of the structure to best fit the models [12]. In particular, characterising self-assembled systems with scattering benefits from using a range of techniques to narrow the scope of possible structures since the process dependent versatility of these materials can cause the nano- and microstructure to vary significantly. Therefore, it is not sufficient to consider only the chemical structure of the compound, but also the environment in which self-assembly takes place. A recent study by Zhang et al. exemplifies the morphological sensitivity of such self-assembled systems across a range of conditions [89]. With changes in molar ratio of the component structures, a range of topologies were accessed including dendritic structures; nanorods aggregated into fractal structures, disordered structures, as well as remaining discrete; and amorphous nanoparticles [89]. Similarly, a change in the solvent allowed a number of morphologies to be accessed (Figure 18), showing that characterisation of a material in a single environment is not necessarily sufficient to narrow the scope of scattering models which are appropriate to apply to a self-assembled material across conditions.

**Figure 19:**
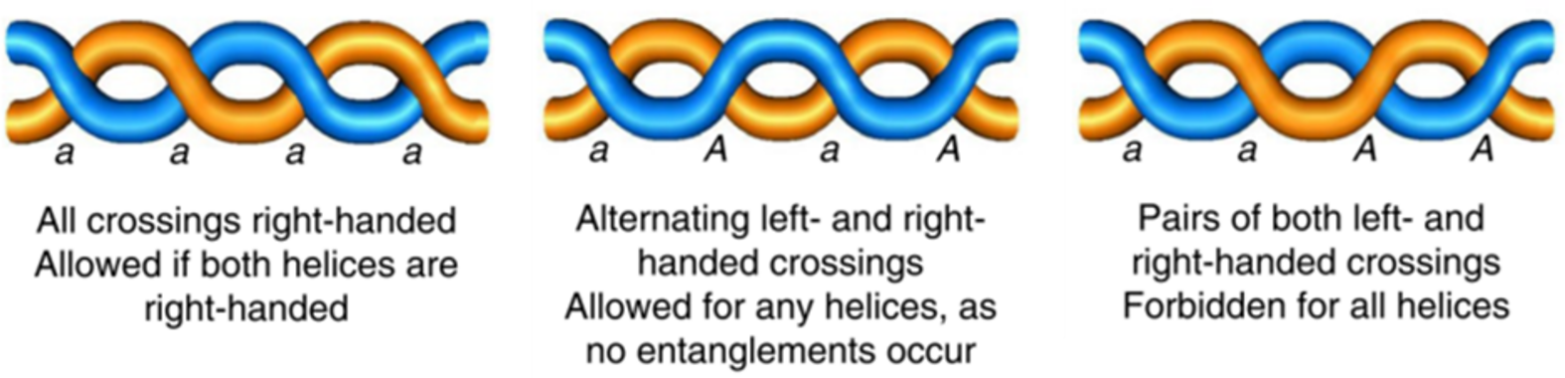
Allowed and forbidden crossings of entangled helices. Figure 19 is from [44] and was adapted by permission from Springer Nature from the journal Nature Chemistry (“Braiding, branching and chiral amplification of nanofibres in supramolecular gels“ by C. D. Jones; H. T. D. Simmons; K. E. Horner; K. Liu; R. L. Thompson; J. W. Steed), Copyright 2019, The Author(s), under exclusive licence to Springer Nature Limited. This content is not subject to CC BY 4.0.

The interpretation of microscopy data can also be aided by utilising a priori knowledge, as demonstrated by Jones et al. The direct images of the self-assembled braids (Figure 9) allows them to be more easily visualised compared to what scattering methods would provide, at the cost of reducing a 3-dimensional structure to a 2-dimensional image which can make it difficult to understand the structure fully. To characterise the topology of the aggregates observed, Jones et al. first identified the helical nature of the discrete fibrils. With this knowledge, the possible aggregate structures were reduced by considering the allowed and forbidden interactions of helices (Figure 19). By utilising mathematical knot theory and the topological constraints imposed by the interaction of helices it was shown that structures composed of two entangled fibrils can only lead to a double helix, and three fibrils must yield a triple-helix or Brunnian braid. The power of applying such a priori knowledge is shown in the case of braids formed of four fibrils. It was shown that there are 354 braids which can result from the entanglement of four fibrils, but only four of these structures have the favourable characteristics that would allow them to form. This reduction in the possible structures formed allows the topology to be assessed much more effectively.

Computational models can also be a key tool used to inform and interpret characterisation data. For example, Hirst et al. predicted the dimensions of a gelator molecule using a computer simulation to rationalise the scattering resulting from self-assembling gelator molecules. The SAXS characterisation of this system indicated the presence of cylindrical structures with a radius of 18 Å. By using a Corey–Pauling–Koltun model, the gelator monomer was predicted to have a length of 20 Å. This evidence suggested that SAXS characterisation could be rationalised as filaments comprising two molecules lying end-by-end across the radius structure. The thickness of the molecule also indicated that 4 molecules could be radially arranged giving rise to the cylindrical topology observed by SAXS [90]. Similarly, Angelerou et al. used molecular dynamics (MD) simulations to model the self-assembled structure of a deoxycytidine derivative. By calculating the radial distribution functions of the MD, the molecular length and fibre diameter were determined, and were in accordance with powder X-ray diffraction analysis. SANS analysis indicated the presence of flexible cylinders with a diameter four times larger than those determined by the MD simulations. This indicated that SANS was probing the complex architecture of bundled fibres as opposed to the individual fibres [91].

**Figure 20:**
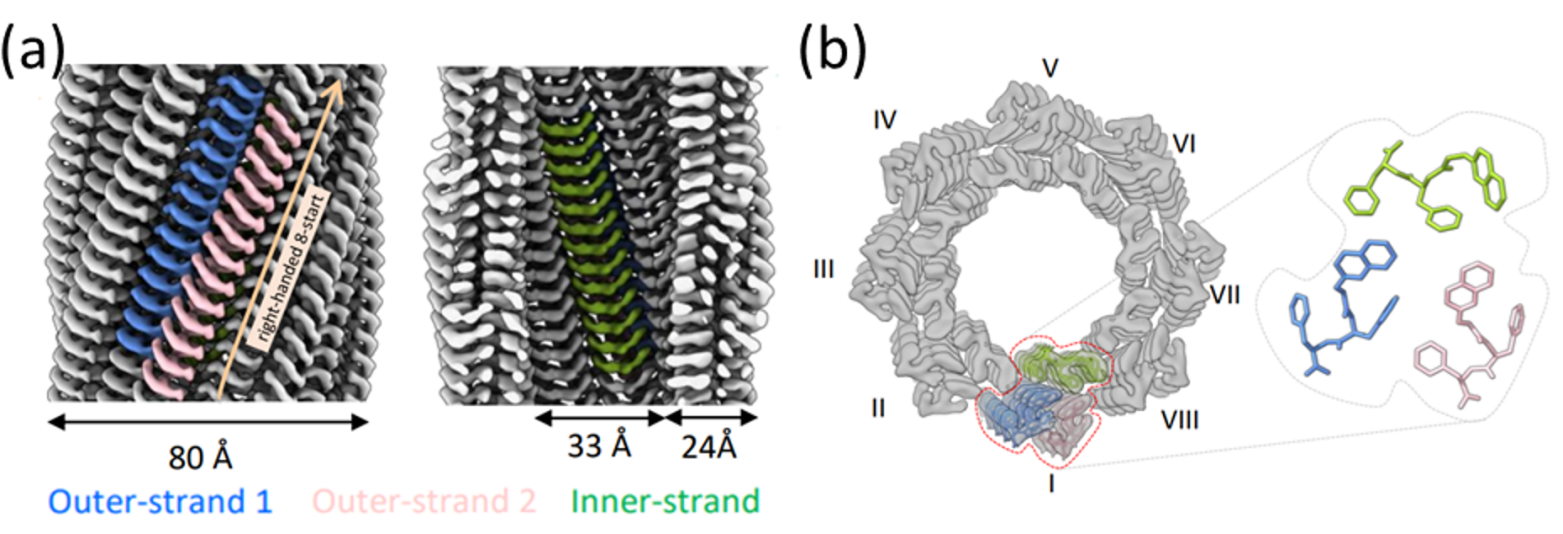
(a) Cryo-TEM density map of self-assembled (ʟ,ʟ)-2NapFF. (b) Computational model fit to cryo-TEM map showing eight protofilaments and conformers making up each trimer unit. Figure 20 was adapted from [92] (© 2024 R. Sonani et al., published by Elsevier, distributed under the terms of the Creative Commons Attribution-NonCommercial-NoDerivs 4.0 International License, https://creativecommons.org/licenses/by-nc-nd/4.0/). This content is not subject to CC BY 4.0.

Recently, following on our groups work with 2NapFF [68], Sonani et al. determined, at near-atomic resolution, cryo-electron microscopy structures resulting from the self-assembly of 2NapFF and 2NapFF. Previously, molecular packing was only predicted from low-resolution scattering data, and so the actual packing remained undetermined. Using Cryo-TEM, the highly-ordered packing and helical symmetry of the 2NapFF self-assembly was directly observed, allowing high-resolution atomic models to be constructed. This showed that the micelle structure arises from eight protofilaments consisting of a trimer of 2NapFF molecules in various conformations (Figure 20) [92].

The cryo-TEM model corroborated previous scattering data, however, this technique was better able to show that packing was driven by burying the aromatic rings in the hydrophobic core. Furthermore, the long-range stiffness of these assemblies could be rationalised by the charge repulsion of the solvent-accessible carbonyl groups. This improved understanding of the structural features which control the self-assembled nanostructure of 2NapFF may be a crucial step to achieving the rational design and modification of these compounds [92]. This cryo-TEM technique provided a resolution that could not be achieved in previous investigations, although such a study relied on the significant a priori knowledge that has resulted from many years of study on these compounds within our group. The understanding achieved in this study was possible only with the plethora of characterisation knowledge afforded by previous microscopy and scattering characterisations. We highlight here that even if a technique may have the capability of elucidating important characteristics of a self-assembled system, a priori knowledge afforded by other techniques is often first required to effectively extract such information.

We have described here some interesting applications of utilising a prior knowledge to better interpret characterisation data. However, there exists a number of methods which can probe supramolecular assemblies to inform characterisation of the nanostructures and microstructures. These include nuclear magnetic resonance (NMR) spectroscopy, infrared spectroscopy, circular dichroism, fluorescence, dynamic light scattering, and rheology among others [11]. Contreras-Montoya et al. provides a prime example of a comprehensive analysis of a system using a broad range of techniques in order to identify and understand the gelation mechanism giving rise to distinct gel morphologies from the same solution [93]. Polarised optical microscopy and SEM could identify the presence of both liquid crystalline and fibrous assemblies, respectively. NMR was compared to a priori X-ray crystallography and diffraction experiments to identify the conformers present in solution. X-ray powder diffraction revealed the thermodynamics prefer a linear conformation while an antisymmetric conformation was kinetically favoured. SANS as corroborated by SEM allowed for the quantification of the structural changes as the various gel morphologies formed. Circular dichroism (CD) was used to confirm the conformational change and emergence of helical or linear self-assembled fibres as observed in SEM. Rheology supported the SANS characterisation by rationalising the change in bulk mechanical properties with the change in the cross-link density. Finally, changes in the CD spectrum over time informed about the kinetics of self-assembly for each of the morphologies. Together, this extensive collection of data allowed the pathway complexity in which complex interactions between slowly equilibrating solution conformers gives rise to very different bulk materials properties to be understood.

As detailed in this section, a comprehensive understanding of complex self-assembled systems cannot be achieved with a single technique alone. However, while such data is essential for a robust characterisation, it can not only be difficult to perform but also difficult to access.

### Characterisation access

One of the major limitations of scattering and microscopy techniques is the facility access. There are a number of synchrotron facilities capable of offering SAXS services including at ANL (USA), DESY (Germany), Diamond Light Source (UK), ESRF (France), Spring-8 (Japan), and SSRL (USA) [94]. Similarly, various facilities offer access to microscopy techniques, the largest including SEMC (USA), Diamond Light Source (UK), and SUSTech (China) [95].

Much like the Diamond Light Source, many beamlines accept proposals from researchers worldwide, granting beamtime on the scientific merit of proposals as determined by a peer-reviewed panel of researchers. The available time at such facilities is limited and in high demand, which can make accessing them difficult, even for established academic researchers [90]. Despite this, access remains inequitable for some researchers due to financial aid often being limited to researchers from the region in which the synchrotron facility resides or are partnered with. With the majority of facilities being limited to North America, Europe, and Eastern Asia, this can lead to increased limitations to synchrotron techniques for those based outside of those regions [96]. Having to travel long distances to access such facilities is often difficult for those who are not able-bodied or for those who have caring responsibilities, and so more local facilities would be more equitable. Laboratory scale SAXS sources are available with a suitably low background allowing for the measurements of low scattering signals, whilst requiring the same amount of material. However, such setups provide a much lower flux than at synchrotron sources, resulting in measurement times increasing from seconds at synchrotrons to hours in the laboratory [94]. While this allows standard, time-averaged processes to be captured, often the kinetics of self-assembly occur at timescales much smaller than laboratory SAXS will probe, limiting many in situ processes to synchrotron SAXS facilities. The cost of laboratory scale SAXS sources are >£350k (as of 2023) for purchase cost alone, without upkeep or technicians to help run the equipment, meaning even these are not accessible to most research groups.

**Figure 21:**
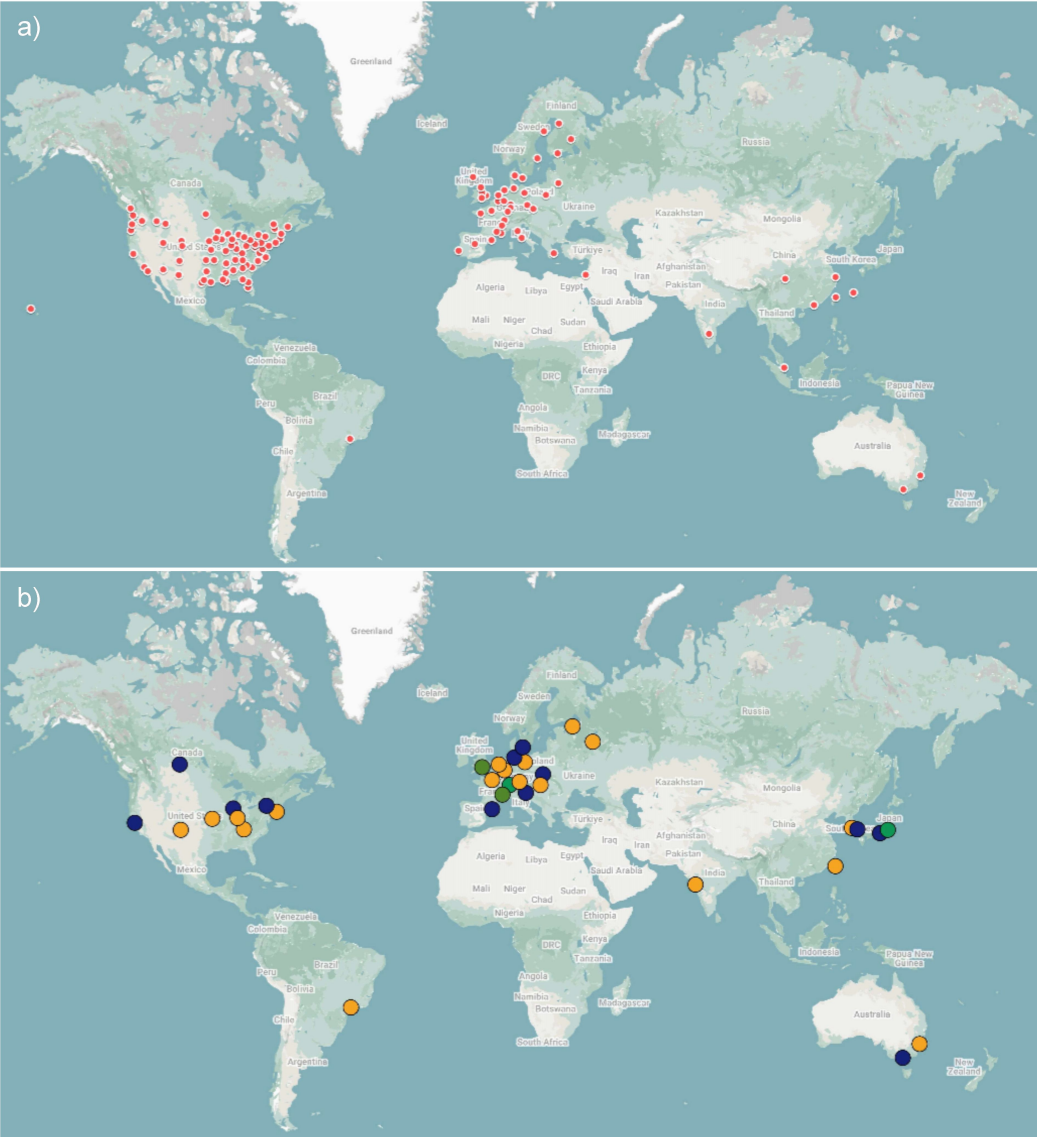
Map showing an incomplete list of global scientific centres providing access to (a) cryo-EM in red and (b) synchrotron neutrons in yellow, synchrotron X-rays in blue, or both in green highlighting the geographic inequity of beamline access [97–99]. Map data ©2024 Google INEGI This content is not subject to CC-BY 4.0.

Despite electron microscopes being more easily distributed than, for example, synchrotron sources and much less expensive for simple instruments; large cryo-EM facilities are all co-located with other core utilized services, creating a high degree of strategic flexibility and support through incorporation of different specialties [95]. However, this significant centralisation provided synchrotron facilities, while essential for the development and access of microscopy and scattering techniques, does not eliminate all barriers which need to be overcome to provide equitable access for scientists who conduct critical research in underserved regions (Figure 21). Despite the availability of remote access options, many samples are not stable enough to travel long distances, and the costs associated may be significant [95]. Therefore, simply providing remote access options for scientists in regions without cryo-EM facilities may result in barriers to techniques critical for material characterisation. The use of microscopy techniques benefits from a network of collaborative centres which facilitate access, sustain innovation, and ultimately broaden the reach of cryo-EM [95]. Therefore, international collaborations are critical to ensuring sufficient access to both scattering and microscopy techniques. Access to such facilities allows users with minimal local instrumentation to gain experience, however, establishing more accessible facilities better enables researchers to perform their own analysis as opposed to remotely accessing these techniques. The necessity for users to process their own data to utilise their unique knowledge instead of being limited to the beamline operator's assessment is discussed later. It has previously been concluded that establishing new facilities better develops the experience of researchers which is necessary to enable such analysis [95]. Subsequently we highlight some of the historic efforts to maximise the access of centralised facilities, and the ongoing efforts to establish new facilities in underserved regions to foster better access for the growing synchrotron communities worldwide.

Neutron facilities in Europe were among the earliest pioneers of the concept of a user programme, providing access to external researchers. This led to the construction of the Institut Laue-Langevin (ILL) as a purpose-built user facility serving a broad community of researchers. Such extensive collaboration laid the foundation for Europe as a dominating force in neutrons science, with 70% of high impact neutron science papers resulting from experiments at European neutron facilities. This has been aided by schemes such as the EU funded Transnational Access programme which significantly increased the use of neutron techniques by providing access to countries without a national neutron facility [100]. Collaborations such as the league of advanced neutron sources (LENS) have proven instrumental in the success of the European neutron community and highlight the need for such extensive partnerships to promote and enhance developing neutron communities.

Substantial collaborative efforts are underway to resolve the limited synchrotron access across the world. The partnership between the ESRF beamline and South Africa established in 2013 was a critical step in growing the community of light source users that could form the core of an African light source (AfLS) community [96]. The growing interest lead to the 1st AfSL conference held at the ESRF in 2015 wherein the importance of developing an AfSL was captured in their first resolution: “Advanced light sources (AdSLs) are the most transformative scientific instruments similar to the invention of conventional lasers and computers” [96]. As part of the roadmap to this goal, the AfSL plan to establish formal partnerships with existing international light sources which could drastically improve access for African scientists. In addition to the AfSL initiative, international collaborations such as the light sources for Africa, the Americas, Asia and Middle East project (LAAAMP) aim to enhance AdSL science across these underserved regions [101].

The work of the AfSL initiative and LAAAMP, among others, highlights that significant efforts are underway to foster light source communities around the world. However, there is still a demand for increased collaboration to serve the need for light source access in these communities. Therefore, we present here that microscopy data is best used in conjunction with scattering techniques, but it is important to note that researchers may not have access to facilities that allow both (or potentially either) characterisation techniques to be utilised. However, in order to gain meaningful and reproducible data from these techniques once access is gained, one must consider how the data is processed and reported.

### Reporting standards for model fitting

Our group has over a decade of experience working with scattering techniques. Over this time, we have acquired substantial knowledge regarding the application and processing of fitting models. For many groups inexperienced with these techniques, data can be fitted by beamline scientists and the best fit models provided to the user. However, this can give rise to problems when interpreting and reporting data. As our understanding of these metrics have improved, so too has the quality of our reporting of scattering data. To improve the application of scattering techniques to the field of self-assembled materials, a more complete reporting of the resulting data is required in publications. Using the knowledge acquired from our experience with scattering data, we describe here some best practices for processing scattering data and reporting fits.

One of the limitations of scattering methods is the complexity of model fitting. Model fitting allows structural features in real space to be interpreted from the q space scattering. Such models describe the scattering that arises from a structure’s topology. A range of models describing various topologies are available, however, solving these often results in multiple solutions [12]. Differing models can result in acceptable fits. This means that a priori knowledge is required in order to identify which models appropriately describe the system. When analysed by a beamline scientist who is unfamiliar with the system, they will have limited a priori knowledge. This can lead to the selection of the “best” model purely on the basis of fitting characteristics such as χ^2^ (a parameter relating to the goodness of fit) and parameter errors. However, if one has a more intimate knowledge of the sample, a number of models (potentially including those with better fitting characteristics) can be rejected on the basis that they do not corroborate other previously observed characteristics of that material. Therefore, we suggest that relying solely on the beamline scientist's interpretation as being the definitive interpretation of the scattering data may lead to poor characterisations. Beamline scientists will often lack crucial information about a self-assembling system which could lead to unrealistic morphologies being naively presented as the best fit to the data, especially when differing models yield similar fits to the data. While their expertise can aid the fitting procedure, without sufficient a priori knowledge on the systems they are modelling, beamline scientists may be limited to selecting the “best” interpretation solely on the basis of the optimisation of goodness of fit parameters. While such parameters are a good indication that a fit reasonably reflects the observed data, relying on them alone without considering the validity of the morphological parameters increases the likelihood that overfit models are reported. It is recommended that data is processed by the user, such that a priori knowledge can be applied to disregard inconsistent fits. For a comprehensive guide on applying a rigorous fitting procedure to scattering data, we recommend a tutorial prepared by McDowall [102].

An additional benefit of processing scattering data within the research group is that it becomes more apparent what information is of importance to those publishing the data. It has been observed across the literature that it is common to report scattering data simply by showing the fit overlayed on the scattering data, and some accepted fitting parameters. This likely arises from a lack of understanding of the model fitting procedure, and an unawareness of the metrics which can be used to determine the quality of a fit. To contrast the differences in reporting we compare an early example of scattering in our group by Colquhoun et al. with a more recent publication by McDowall et al. which benefitted from significant scattering knowledge in the group.

**Figure 22:**
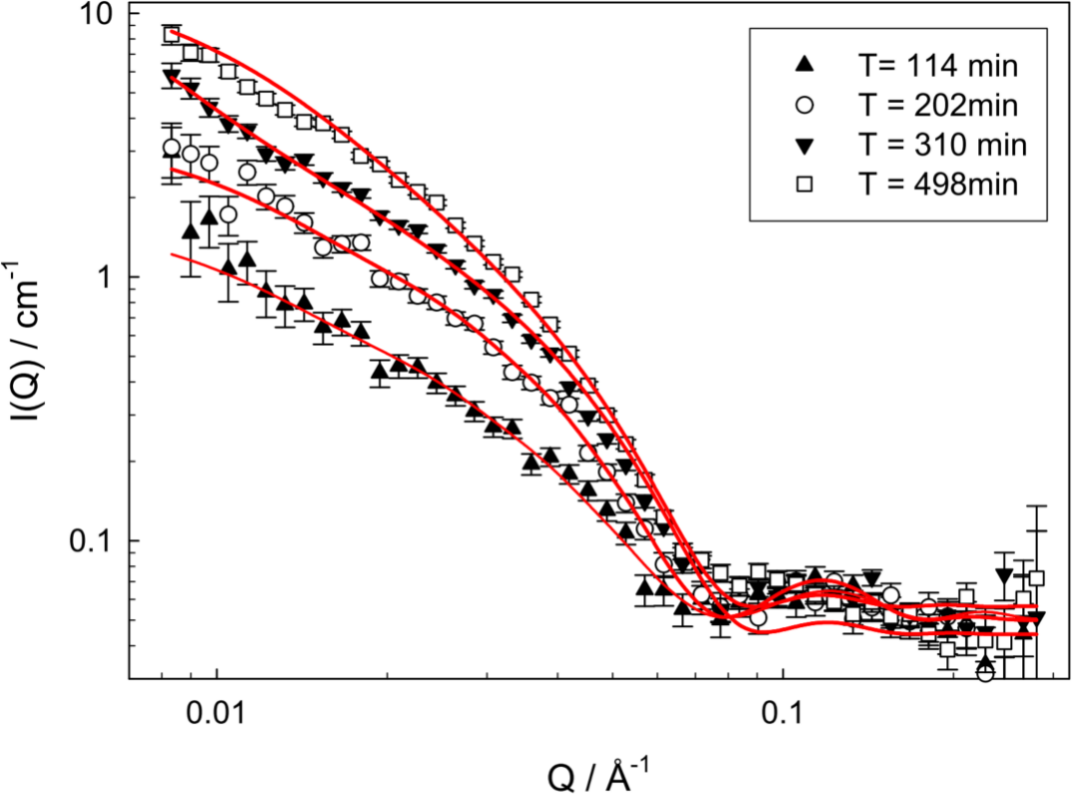
SANS at a range of times. Solid lines are fits to a hollow cylinder model (*T* = 114 min and *T* = 202 min) or fits to a flexible cylinder model (*T* = 310 min and *T* = 498 min). Figure 22 was reproduced from [103] (“The effect of self-sorting and co-assembly on the mechanical properties of low molecular weight hydrogels” © 2014 C. Colquhoun et al., published by The Royal Chemical Society, distributed under the terms of the Creative Commons Attribution 3.0 International License, https://creativecommons.org/licenses/by/3.0).

The work by Colquhoun et al., like many examples in the literature, reported their scattering data with very limited information. The paper itself details the fitting parameters of the accepted model, with a supplemental figure simply overlaying the scattering plot with the model fit (Figure 22) [103]. While this indicates that cylindrical models appear to be suitable, it does not adequately indicate why, or even if this is the most acceptable model.

**Figure 23:**
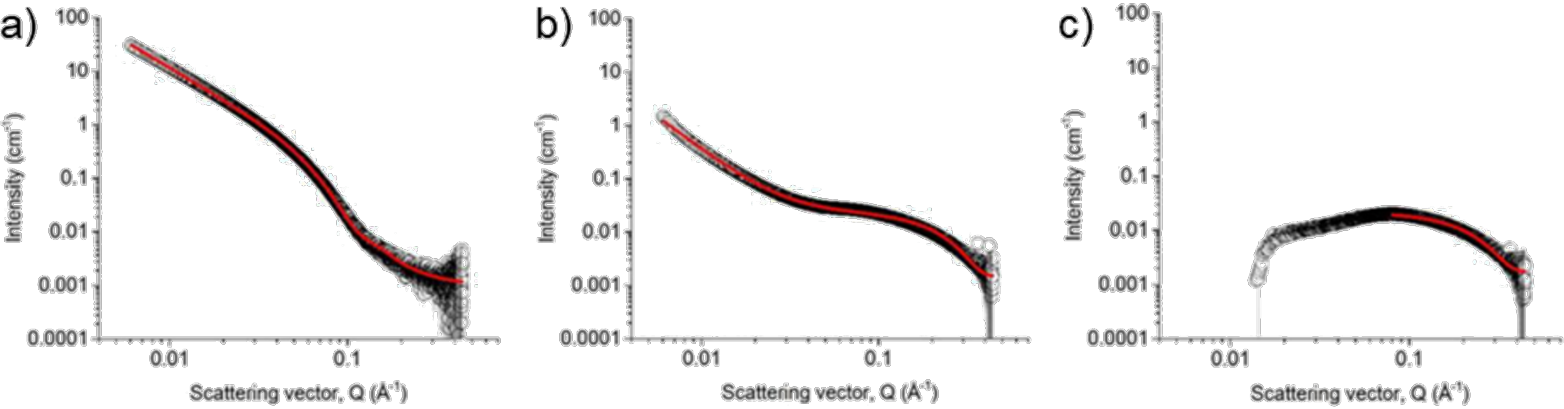
SAXS data of 5 mg/mL alanine-functionalised perylene bisimide (PBI-A) in 20 v/v % MeOH at pH (a) 2; (b) 6; and (c) 10. Figures show the scattering vector vs scattering intensity (open black circles) with the corresponding model fits (solid red lines). Figure 23 was adapted from [61] (© 2020 D. McDowall et al., published by Wiley-VCH GmbH, distributed under the terms of the Creative Commons Attribution 4.0 International License, https://creativecommons.org/licenses/by/4.0).

In contrast, McDowall et al. comprehensively describes their acceptance criteria by highlighting a few decision criteria, as well as including more detailed fitting parameters. Firstly, the types of models investigated were described and rationalised. A priori knowledge informed about the likely formation of 1D fibres and worm-like micelle structures, therefore cylindrical models were chosen as the most appropriate rationalisation. The inclusion of spherical models to represent smaller aggregates and power law models to represent larger network structures were also rationalised. Secondly, it is made clear that the complexity of the models was increased only as required. Single models with fewer parameters were investigated first, followed by more complex single models, and ultimately combined models indicating the co-existence of structures. Adding further parameters to a model will always reduce the χ^2^, which can ultimately result in overfitting. Therefore, a model with fewer parameters that fits the data well is preferable, as parameters from overfit models are less meaningful. Thirdly, the selection criteria are established, providing a clear rational for the most appropriate model. While χ^2^ is a useful metric for evaluating the quality of a model, differentiating between two similar models on this basis alone was insufficient. In such a case, the lowest χ^2^ was accepted unless regions of the fit were not representative of the dataset. Finally, in addition to visually comparing the model to the scattering data (Figure 23), the parameters and accepted metrics are tabulated to give a complete description of the model (Table 4). This includes the errors of the parameter fit, which is a good indication of how well the parameters represent the observed scattering [61].

**Table 4:** Model fitting results for of 5 mg/mL PBI-A in 20 v/v % MeOH.^a^

model	pH	length (nm)	Kuhn length (nm)	cylinder radius (nm)	sphere radius (nm)	power law	reduced χ^2^

flexible cylinder with a polydispersity of radius 0.2 + power law	2	680^a^	5.9 ± 0.08	3.2 ± 0.01	–	2.7 ± 0.00	3.4
sphere + power law	6	–	–	–	1.0 ± 0.00	2.6 ± 0.01	1.0
sphere	10	–	–	–	1.0 ± 0.00	–	<1

^a^No fitting error available. Length increased to an unrealistically large number with further fitting. Length set to 680 nm and a power law added to capture the intensity at low Q. Table 4 was reproduced from [61] (© 2020 D. McDowall et al., published by Wiley-VCH GmbH, distributed under the terms of the Creative Commons Attribution 4.0 International License, https://creativecommons.org/licenses/by/4.0).

We believe that the data reported by McDowall et al. is an exemplary standard of scattering data reporting and subsequently recommend the application of such a robust procedure when modelling structures with scattering techniques and reporting the subsequent results. Not only would this make fitting more reproducible but would make clearer why a model may have previously been erroneously disregarded as further characterisation data is acquired.

Similar to the reporting of SAS, there are a number of parameters which can significantly change the results of characterisation using microscopy methods which often go unreported. As previously discussed, there exist a few methods to prepare samples for microscopy. The high-vacuum environment required by SEM and TEM demands that samples be analysed in the dry state. The desiccation procedure will alter the material structure, resulting in the quantification of an unrepresentative hydrogel network [37]. The coating procedure that follows this will irreversibly alter the surface of the hydrogel which can conceal finer structural details [104]. For cryo-EM analysis, there are a various freezing regimes which cool the samples at different rates. These include liquid nitrogen slush (LNS), liquid ethane, and high-pressure freezing (HPF). The quality of cryo-EM data requires a sufficient large cooling rate to avoid the formation of hexagonal ice crystal formation which will result in a change in the hydrogel structure. Aston et al. have previously demonstrated the significant impact that the choice of the cryo-fixation technique has on both the macrostructure and microstructure, with the macropore size, micropore size, and number of micropores varying by an order of magnitude by changing the cryo-fixation alone [14]. The dependence of the quantification on the choice of fixation technique shows that any results must be reported along with the preparation regime in order to be reproducible, which is sometimes lacking in the literature.

**Figure 24:**
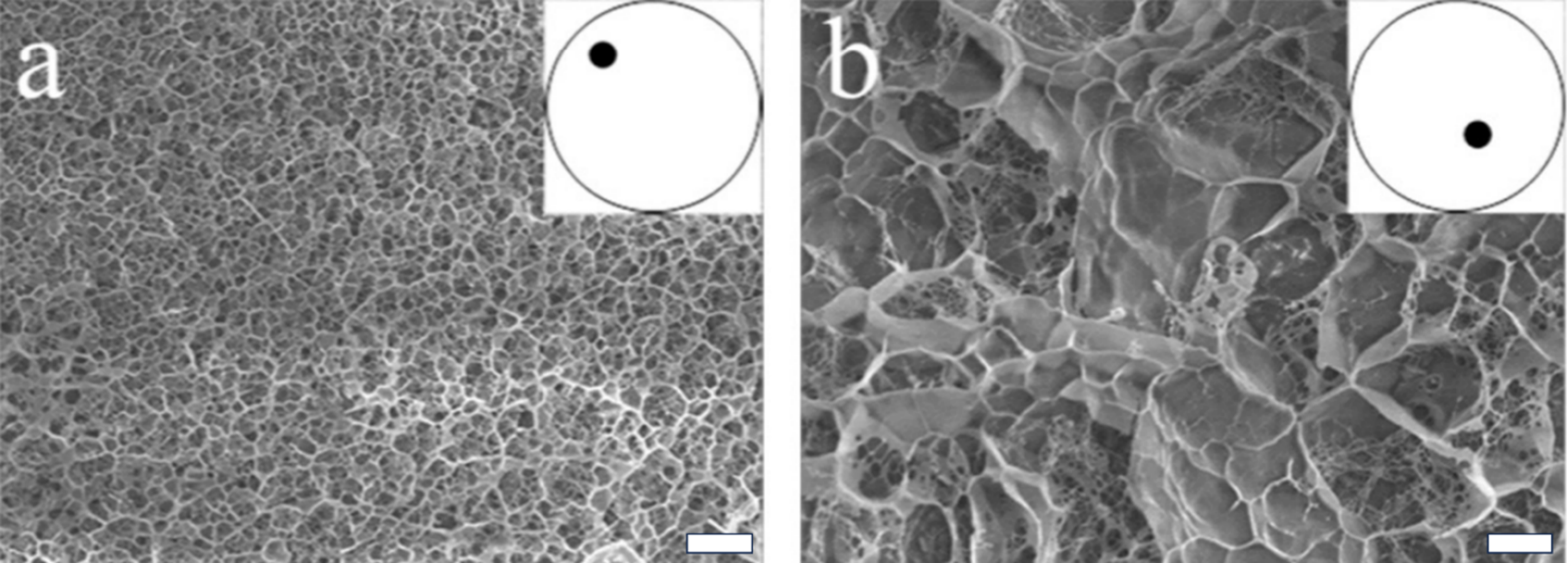
Cryo-TEM sample prepared using plunge freezing in liquid nitrogen slush and sublimed for 30 minutes: (a) 25 µm from sample edge and (b) >200 µm from the sample edge. Scale bar represents 1 µm. Figure 24 was adapted from [14], European Polymer Journal, vol. 82, by R. Aston; K. Sewell; T. Klein; G. Lawrie; L. Grøndahl, “Evaluation of the impact of freezing preparation techniques on the characterisation of alginate hydrogels by cryo-SEM”, pages 1-15, Copyright (2016), with permission from Elsevier. This content is not subject to CC BY 4.0.

Furthermore, the variability arising from the choice of imaging location within a single vitrified sample was also demonstrated, wherein imaging the edge of a vitrified hydrogel compared to the centre of the sample revealed pores which differed by an order of magnitude in size (Figure 24) [14].

Based on their findings Aston et al. provided recommendations for the reporting of microscopy methods for the quantification of supramolecular hydrogels. These include: providing a description of the hydrogel preparation, reporting the sublimation time, reporting the type of coating used (in addition to analysing the uncoated material to ensure the coating process does not alter the hydrogel structure), specifying the imaging location on the sample, and reporting the image magnification used to determine the structural features [14]. The process-dependent nature of supramolecular materials makes them particularly sensitive to changes in procedures not only in their synthesis, but also their characterisation. In our opinion, to overcome the current unpredictability afforded by these materials, there must first be sufficient, robust, and reproducible data across such materials to identify the underlying structures and their subsequent properties. This can only be achieved by the collective adoption of reporting standards such as those discussed here.

Even when a robust experimental process is implemented, it is important that the data processing is performed while taking steps to avoid bias in data sampling. Applying a priori knowledge has been shown to be an integral part of fully characterising data, however, it is important that such insights are applied rationally. As shown in Figure 24, depending on the region of the sample imaged, a wide range of structures can be captured. It is possible therefore that data in agreement with a priori results are reported while disregarding those structures which deviate from the expected morphology. It is important to note that biased data, including that resulting from conscious and unconscious bias, is difficult to recognise. Such data will vary in accuracy and precision so there is no simple way to identify the skewing of data. This limitation is not unique to the characterisation of materials, and solutions have been considered for other applications such as in the measurement of mitochondria in TEM [105]. Not all recommendations such as analysing samples blind are practicable for highly variable supramolecular systems since a degree of a priori knowledge may be required to effectively interpret the data as previously discussed. However, there are steps that can be taken to improve the processing of microscopy data. Analysis should be performed using a well-documented methodology to minimise bias in processing. Furthermore, this method should be applied to a large sample of measurements [105]. Not only does this aid in ensuring the quality of characterisation, but it should also lead to a much more reproducible procedure which will allow for a more useful comparison between systems.

## Conclusion

We have provided a rationale for using a combination of microscopy and scattering techniques to characterise self-assembled systems and caution that relying on a single method of characterisation may not provide a complete description of a system. Moreover, these techniques, especially scattering techniques, require a reasonable degree of a priori knowledge to effectively identify nanostructure and microstructural features. We do note, however, that the access to scattering techniques can be limited, especially to scientists with little access to synchrotron facilities. We once again stress the importance of providing a complete report on the fitting procedures used to characterise systems with scattering techniques, following a robust fitting procedure aided with a priori knowledge.

## Data Availability

Data sharing is not applicable as no new data was generated or analyzed in this study.
